# GC-B Deficient Mice With Axon Bifurcation Loss Exhibit Compromised Auditory Processing

**DOI:** 10.3389/fncir.2018.00065

**Published:** 2018-08-29

**Authors:** Steffen Wolter, Dorit Möhrle, Hannes Schmidt, Sylvia Pfeiffer, Dennis Zelle, Philipp Eckert, Michael Krämer, Robert Feil, Peter K. D. Pilz, Marlies Knipper, Lukas Rüttiger

**Affiliations:** ^1^Department of Otolaryngology, Head and Neck Surgery, Molecular Physiology of Hearing, Tübingen Hearing Research Centre, University of Tübingen, Tübingen, Germany; ^2^Interfaculty Institute of Biochemistry, University of Tübingen, Tübingen, Germany; ^3^Department of Animal Physiology, University of Tübingen, Tübingen, Germany; ^4^Department of Otolaryngology, Head and Neck Surgery, Physiological Acoustics and Communication, Tübingen Hearing Research Centre, University of Tübingen, Tübingen, Germany

**Keywords:** Npr2, olivocochlear system, cochlear efferents, development, ABR, DPOAE, acoustic startle, PPI

## Abstract

Sensory axon T-like branching (bifurcation) in neurons from dorsal root ganglia and cranial sensory ganglia depends on the molecular signaling cascade involving the secreted factor C-type natriuretic peptide, the natriuretic peptide receptor guanylyl cyclase B (GC-B; also known as Npr2) and cGMP-dependent protein kinase I (cGKI, also known as PKGI). The bifurcation of cranial nerves is suggested to be important for information processing by second-order neurons in the hindbrain or spinal cord. Indeed, mice with a spontaneous GC-B loss of function mutation (*Npr2^cn/cn^*) display an impaired bifurcation of auditory nerve (AN) fibers. However, these mice did not show any obvious sign of impaired basal hearing. Here, we demonstrate that mice with a targeted inactivation of the GC-B gene (Npr2*^lacZ/lacZ^*, GC-B KO mice) show an elevation of audiometric thresholds. In the inner ear, the cochlear hair cells in GC-B KO mice were nevertheless similar to those from wild type mice, justified by the typical expression of functionally relevant marker proteins. However, efferent cholinergic feedback to inner and outer hair cells was reduced in GC-B KO mice, linked to very likely reduced rapid efferent feedback. Sound-evoked AN responses of GC-B KO mice were elevated, a feature that is known to occur when the efferent axo-dendritic feedback on AN is compromised. Furthermore, late sound-evoked brainstem responses were significantly delayed in GC-B KO mice. This delay in sound response was accompanied by a weaker sensitivity of the auditory steady state response to amplitude-modulated sound stimuli. Finally, the acoustic startle response (ASR) – one of the fastest auditory responses – and the prepulse inhibition of the ASR indicated significant changes in temporal precision of auditory processing. These findings suggest that GC-B-controlled axon bifurcation of spiral ganglion neurons is important for proper activation of second-order neurons in the hindbrain and is a prerequisite for proper temporal auditory processing likely by establishing accurate efferent top-down control circuits. These data hypothesize that the bifurcation pattern of cranial nerves is important to shape spatial and temporal information processing for sensory feedback control.

## Introduction

Sensory axons of dorsal root ganglia (DRG) and cranial sensory ganglia (CSG) including cochlear spiral ganglion neurons (SGN) undergo a T-shaped branching (bifurcation) before the formation of collaterals that synapse onto second-order neurons in the spinal cord or hindbrain. The importance of this bifurcation of cranial sensory axons is currently not understood and it is elusive whether it is beneficial or even required in vertebrates to transmit the sensory information to the second-order neurons in the hindbrain or spinal cord. It has been shown that axons of the DRG, CSG, or SGN fail to bifurcate in the absence of guanylyl cyclase B (GC-B) (Schmidt et al., 2007; Lu et al., 2014; Ter-Avetisyan et al., 2014). GC-B is a transmembrane receptor guanylyl cyclase that upon binding of its ligand C-type natriuretic peptide (CNP), which is expressed dorsally along the length of the embryonic neural tube (Potter et al., 2006; Schmidt et al., 2009), activates a cGMP signaling cascade involving cGMP-dependent protein kinase Iα (cGKIα) (Schmidt et al., 2002). The lack of bifurcation of fibers from the vestibulocochlear (VIII) sensory nerve due to a point mutation in the *GC-B* gene of *Npr2^cn/cn^* mutant mice led to only subtle changes in auditory function with normal basic hearing and vestibular function. Deficits were found in the tonotopic organization typical of central auditory circuits (Lu et al., 2014). This first piece of evidence indicates that auditory signals are still transmitted and basic hearing function is preserved in mutant mice despite the loss of axon bifurcation of auditory nerve (AN) fibers during development (Lu et al., 2014). Lu et al. (2014) showed that bifurcation deficits of SGNs went along with disrupted tonotopic organization of AN fiber terminals in all divisions of the cochlear nucleus (CN) [anterior ventral cochlear nucleus (aVCN), posterior ventral cochlear nucleus (pVCN), and dorsal cochlear nucleus (DCN)]. Furthermore, beside the disrupted tonotopic organization, also the convergence of SGN inputs to multipolar cells in the CN may be altered in GC-B deficient mice (Lu et al., 2014): T-Stellate cells from VCN displayed a limited dynamic range of response growth to gradual increasing electrical stimulation, indicating that integration and encoding of sound is disturbed. Strikingly, the multipolar cells in the VCN have been proposed as the interneurons for the cholinergic efferent feedback to the cochlear outer hair cells (OHCs) thereby controlling cochlear amplification via the medial olivocochlear bundle (MOC) (De Venecia et al., 2005). In fact, planar multipolar neurons in the VCN have been shown to make projections to the ventral nucleus of the trapezoid body where they were observed terminating on MOC neurons (Darrow et al., 2012). The cochlear efferent feedback system in GC-B mice has not yet been examined. In the present study, a closer investigation of a *GC-B* knockout model (*Npr2^lacZ/lacZ^*) that was created by insertion of a *lacZ* expression cassette into exon 1 of the *GC-B* gene (Ter-Avetisyan et al., 2014) revealed a mild, but significant, elevation in hearing thresholds in *GC-B* deficient mice, linked to weaker mechanical amplification of acoustic signals by OHCs and reduced cholinergic feedback to hair cells, that themselves were normally developed. In our study, the measurement of auditory function, including amplitudes and latencies of supra-threshold auditory brainstem response (ABR) wave fine structure, auditory steady state responses (ASSR), prepulse inhibition (PPI) of the acoustic startle response (ASR), and ipsilateral adaptation of distortion product otoacoustic emissions (DPOAEs) suggest a role of axon bifurcation for the establishment of feedback control and precise temporal auditory processing.

## Materials and Methods

### Animals and PCR Genotyping

*Npr2-lacZ* mice were generated from an ES-cell line of 129/Ola background by injection into C57BL/6J blastocysts, and selecting chimeras that transmitted the mutant *Npr2-lacZ* gene. These were identified by mating *Npr2-lacZ/+* males to C57BL/6J females. Backcrossing was performed for at least five generations before the mouse line was conveyed to pool breeding to generate animals for the experiments. Genotyping procedures of mutant alleles were conducted as described before (Ter-Avetisyan et al., 2014). For hearing measurements, behavioral experiments and molecular analysis, adult 1–6 month old mice of either sex were used in this study. For behavioral experiments, a second group of 2–4 month old mice was recruited. Mice were anesthetized for hearing tests using a mixture dosed 5 ml/kg body weight (75 mg/kg body weight ketamine hydrochloride, Ketavet, Pharmacia Pfizer, Karlsruhe, Germany; 5 mg/kg body weight xylazine hydrochloride, Rompun 2%, Bayer, Leverkusen, Germany; 0.2 mg/kg body weight atropine sulfate, Atropinsulfat, B.Braun, Melsungen, Germany; diluted with water, Ampuwa, Fresenius KABI, Bad Homburg, Germany). The level of anesthesia was monitored by heart rate, breathing rate and reflex tests for toe-pinch, eye lid and cornea. Anesthesia was supplemented with maximally one third of the initial dose if needed. Animals were housed in the animal care facility of the institute, where noise levels did not exceed 50–60 dB sound pressure level (SPL). Mice were held in groups of one (only fighting males) to five in standard Macrolon cages containing nesting material under a 12-h light–dark schedule (lights on at 7 am) and received food and tap water *ad libitum*. The cages were in an air-conditioned room with the temperature set at 24 ± 1°C and the humidity held at 60 ± 5%. Animal care, procedures, and treatments were performed in accordance with institutional and national guidelines following approval by the University of Tübingen, Veterinary Care Unit, and the Animal Care and Ethics Committee of the regional board of the Federal State Government of Baden-Württemberg, Germany (Reference No. HN 3/14), and followed the guidelines of the EU Directive 2010/63/EU for animal experiments.

### Hearing Measurements

Auditory brainstem responses, DPOAEs, and ASSRs were recorded in a soundproof chamber as described previously (Möhrle et al., 2016, see also **Figure 2F**). In short, ABR thresholds were determined with click (100 μs), noise burst (1 ms, random phase frozen noise), or pure tone stimuli (3 ms, including 1 ms cosine squared rise and fall envelope, 2–32 kHz). OHC function was assessed by determining thresholds and growth behavior of the cubic (2^∗^ f_1_ – f_2_) DPOAE input-output (I-O) functions for L_1_ = -5–65 dB SPL for sinusoidal primary tones f_1_ and f_2_, with f_2_ = 1.24^∗^ f_1_ and L_2_ = L_1_ + 10 dB. ASSRs were measured with amplitude-modulated sinusoidal stimuli using a 11.3 kHz carrier and modulation frequencies between 64 and 2048 Hz with one step per octave. At a fixed modulation frequency of 512 Hz at 40 dB above threshold [dB sensation level (SL)], responses to modulation depths between 0% (unmodulated), and 0.78% to 100% modulation indices (in half-octave steps) were recorded. For I–O functions the carrier level ranged from -10 to 60 dB SL.

#### MOC-Efferent Adaptation of DPOAEs

In GC-B wild type (GC-B WT), GC-B heterozygous (GC-B HET) and GC-B knockout (GC-B KO) mice the ear was exposed to the two stimulus tones (primaries) with the frequencies f_2_ = 11.3 kHz and f_1_ = 9.11 kHz for primary 1 and primary 2, respectively, to elicit DPOAEs in the ipsilateral ear. Primaries were switched on synchronously and remained on constantly for 100 ms. Stimulus onset and offset contained cosine-shaped ramps of 2 ms length. Stimuli were presented 128 times with 500 ms recording intervals (2 presentations per second).

Primaries were presented in combinations of phase-varied pairs of stimulus phases rotated by 90° (f_1_) and 180° (f_2_) for each subsequent presentation (primary tone phase variation) to cancel the primary components in the response of either four summated single presentations (Whitehead et al., 1996; Dalhoff et al., 2015). Successful reduction of the DPOAE amplitudes could be observed when elicited by f_1_ stimulus SPLs ranging from 60 to 70 dB SPL (Kujawa and Liberman, 2001).

The single measurements (128 sweeps) were band-pass filtered (1/8 octave) around the DPOAE frequency, digitally amplified (40 dB), averaged, and Hilbert-transformed to extract the envelope of the 2^∗^ f_1_ – f_2_ DPOAE response in the time signal (SingleSweep custom software, University of Tübingen, ANSI C-code based on LabWindows-CVI, National Instruments, Austin, TX, United States).

The DPOAE amplitude was then analyzed using Microsoft Excel 2016 (Microsoft Cooperation, Redmond, WA, United States) software. Amplitude values were normalized to the noise plateau at the end of the recording (120 ms) and presented in dB. Adaptation (Δ_amp_) was defined as the difference between the maximum amplitude in a 10 ms time window at the non-adapted onset of the response (3–13 ms, amp_max_) and the adapted average amplitude at 80–100 ms (amp_adapt_), again given in dB. In a first step, amplitude values amp_max_ and Δ_amp_ were compared for GC-B WT and GC-B HET mice. Because of small *n*-numbers to gain statistical power and since both genotypes did not differ in respect to the measured parameters and both genotypes have no SGNs bifurcation deficit (Ter-Avetisyan et al., 2014), both groups were combined to make a solid control group for the GC-B KO mice. Statistical trends for smaller DPOAE amplitudes (amp_max_) and adaptation (Δ_amp_) in GC-B KO mice were analyzed by one-sided Student’s *t*-test, subsequently certified by a resampling procedure (Bootstrapping statistics) to control for random statistical outcomes that may be confounded by small *n*-numbers. Experimental data were rearranged in 20 repetitions each with 10,000 resamplings and analyzed for randomness of *p*-values. A *p*-value smaller 0.05 was considered statistically significant.

#### ABR Waveform Analysis

Auditory brainstem response waveforms were analyzed for consecutive amplitude deflections (waves), with each wave consisting of a starting negative (n) peak and the following positive (p) peak. Wave latencies were defined by the onset timing (negative peak) of each corresponding wave. Peak amplitudes and latencies of ABR waves SP, I, II, III, and IV were extracted and defined as wave SP: SP_n_–SP_p_ (0.4–0.9 ms); wave I: I_n_-I_p_ (0.85–1.9 ms); wave II: II_n_-II_p_ (1.45–3.55 ms); wave III: III_n_-III_p_ (2.55–4.6 ms); wave IV: IV_n_-IV_p_ (3.15–6.05 ms). A customized computer program (Peak, University of Tübingen) was used to extract ABR peak amplitudes and latencies based on these definitions. From the extracted peaks, ABR peak-to-peak (wave) amplitude and latency growth functions were calculated for individual ears for increasing stimulus levels. All ABR wave amplitude and latency growth functions were normalized with reference to the ABR thresholds (from -10 dB to a maximum of 70 dB above threshold for wave amplitudes and from 0 dB to a maximum of 70 dB above threshold for wave latencies).

### Acoustic Startle Response and Prepulse Inhibition

#### Subjects for ASR Test

Startle response was tested on five GC-B WT (two males, three females) and seven GC-B KO (four males, three females) adult mice. Preliminary analysis showed no significant influence of age group (2 or 4 month old ANOVA *F*-test F<1) or sex (ANOVA *F*-test F<1) on startle to standard 105 dB stimuli on day 1. Genotypes differed in weight (WT: 20–33 g, KO: 10–20 g). Also weight did not influence startle amplitude (Pearson correlation *R*^2^ = 0.016, *p* > 0.6) and all addressed parameters were balanced between genotypes. Therefore, the statistical analysis was performed for factor genotype without further subgrouping. Since the statistical results did not reveal any difference between WT and HET mice, and the GC-B HET group was small for behavioral data (*n* = 4), they are not included in the presented results. The mice were adapted to the colony room of the startle measuring facility for 14 days before testing began. Testing took place during the light period (8:30 am to 15:30 pm).

#### Apparatus for ASR Test

Startle responses were measured inside a sound attenuated chamber by a movement sensitive piezo accelerometer platform (Startle-Messsystem, University of Tübingen). Movement-induced voltage changes were amplified and filtered (Low-Pass: 150 Hz; Piezo-Amp System, University of Tübingen) and then digitized with 1 kHz (DAP1200e in a standard personal computer; Microstar, Bellevue, WA, United States). Startle amplitude was calculated as the difference between peak-to-peak voltage during a time window of 50 ms after stimulus onset and peak-to-peak voltage in the 50 ms time window before stimulus onset. Stimuli and a continuous 31 dB SPL broadband background noise were produced by a digital signal processing controlled system (Elf-Board with Siggen Software; Medav, Uttenreuth, Germany), amplified and emitted by a loudspeaker (Visaton HTM 5.6, Haan, Germany). Stimuli for all experiments had an intertrial interval of 15 s. Startle stimuli were 20 ms broadband white noise stimuli of 105 dB SPL unless otherwise noted. Prepulses were white noise stimuli. Mice were placed in a wire mesh test cage (5 cm × 8.5 cm × 5.5 cm) with an aluminum floor, inside the sound attenuated chamber (inside measure: 70 cm × 50 cm × 40 cm). The chamber was illuminated by a white 5 W cold light bulb.

#### Procedure for ASR Test

Mice were allowed to adapt for 5–10 min to the testing environment without stimulation 2 days before testing began. On each test day, mice were allowed to adapt for 5 min, then they received 20 startle stimuli for 5 min with 105 dB SPL, which were discarded from the data set. Thereafter, the mice received test stimuli in pseudorandom order.

##### Startle input–output function

In the first experiment, mice received 120 stimuli, 24 each of 75, 85, 95, 105, and 115 dB SPL.

##### Startle stimulus duration

Startle stimuli with a duration of 0.5, 1, 2, 4, 5, 6, 7, or 8 ms were given 20 times. Startle stimuli with a duration of 20 ms were presented 60 times.

##### Prepulse and lead time

Prepulses with 65 dB SPL were presented 3, 6, 12, 25, 50, 100, 200, or 400 ms before the startle stimulus. Prepulse duration was 20 ms if the lead time was >20 ms, or they lasted until startle stimulus began. Each prepulse lead time was presented 20 times. The control startle stimulus without prepulse was presented 60 times.

##### Gap PPI

A constant background noise of 65 dB SPL was given. As prepulses, gaps with a lead time of 50 ms were presented. The gaps had durations of 1.5, 5, and 50 ms. The noise in the gap had a SPL of 31 dB, and in addition 45 and 55 dB for the durations 5 and 50 ms. Each gap type was presented 20 times, the control startle stimulus without prepulse gap was presented 40 times.

#### Statistical Analysis of ASR

The startle responses were averaged for each day, stimulus condition, and mouse. Parametric statistics were then calculated with these averages. For the prepulse effect (prepulses and gaps), the mean response amplitude for each prepulse condition (ASRPP) and mouse was first calculated relative to the startle stimulus alone (ASR0) condition:

(1) Relative ASR =(ASRPP−ASR0)/ASR0

A negative Relative ASR is then referred to as PPI (given in percent). Positive Relative ASR describes prepulse facilitation. For the effect of startle stimulus duration, response amplitudes for each duration (ASRDur) and mouse were calculated relative to the response to 20 ms (ASR20) lasting startle stimuli:

(2) Relative ASR =ASRDur/ASR20

Differences for prepulse and gap measures and startle stimulus duration were statistically compared using the relative measurements. Statistical tests were done with JMP (SAS institute, version 13). Significance was defined as *p* < 0.05. Error bars represent standard error of the mean (SEM).

### Immunohistochemistry

#### Tissue Preparation

For immunohistochemistry, cochleae were isolated, prepared, cryosectioned in 10 μm slices, and mounted on SuperFrost^∗^/plus microscope slides at -20°C as previously described (Knipper et al., 1996, 1998, 2000).

For whole-mount immunohistochemistry, the temporal bone of mature mouse was dissected on ice, fixed and stained as described (Duncker et al., 2013). Mouse cochlear sections were stained as described (Tan et al., 2007). For X-gal staining in whole-mount preparations anti-β-galactosidase monoclonal antibody was used (mouse, 1:100, Promega, catalogue number Z3781, Madison, WI, United States). For hair cell phenotyping, antibodies directed against C-terminal-binding protein 2 (CtBP2/RIBEYE, rabbit, 1:1500, American Research Products, catalogue number 10-P1554, Waltham, MA, United States), otoferlin (mouse, 1:100, LifeSpan Biosciences, catalogue number LS-C153337, Seattle, WA, United States), anti-vGlut3 (rabbit, 1:100, Synaptic Systems, catalogue number 135 203, Göttingen, Germany), potassium voltage-gated channel subfamily KQT member 4 (KCNQ4, mouse, 1:50, StressMarq Biosciences, catalogue number SMC-309D, Victoria, BC, Canada) and prestin [rabbit, 1:3000, Squarix Biotechnology, Marl, Germany (Weber et al., 2002)], were used. For labeling of afferent/efferent innervation of the inner hair cells (IHCs) and OHCs, antibodies directed against choline acetyltransferase (ChAT, rabbit, 1:100, Millipore, catalogue number AB5042, Temecula, CA, United States, **RRID**:AB_91650), Synaptobrevin (vesicle-associated membrane protein isotype 2, VAMP2, mouse, 1:200, Synaptic Systems, catalogue number 104211, Göttingen, Germany, **RRID**:AB_887811), large conductance Ca^2+^-activated K^+^ channel (BK, rabbit, 1:400, Alomone Labs, catalogue number APC-021, Jerusalem, Israel, **RRID**:AB_2313725), and Neurofilament 200 (Nf200, mouse, 1:8000, Sigma-Aldrich, catalogue number N0142, St. Louis, MO, United States, **RRID**:AB_477257) were used.

Primary antibodies were detected using appropriate Cy3- (1:1500, Jackson Immuno Research Laboratories, West Grove, PA, United States) or Alexa488-conjugated secondary antibodies (1:500, Invitrogen Molecular Probes, Paisley, United Kingdom). For double labeling studies, pairs of antibodies were simultaneously incubated for identical time periods. Sections were viewed by an BX61 Olympus microscope as previously described (Zampini et al., 2010). Cochlear sections were imaged by z-stacking, three dimensionally deconvoluted (ADVMLE, CellSens, Olympus, Hamburg, Germany) and displayed as maximum intensity projection over z.

Immunofluorescent staining of embryonic tissues were performed as described previously (Schmidt et al., 2007; Schmidt and Rathjen, 2011). The following primary antibodies were used in combination with appropriate fluorophore-conjugated secondary antibodies: rabbit anti-β-gal (1:20000; Cappel) and guinea pig anti-cGKIα (1:25000) to the N-terminal region of mouse cGKIα (amino acid residues 2–89) (Ter-Avetisyan et al., 2014).

Immunohistochemical stainings of cochlear sections were quantified by integrating density values of color pixels for each single specimen using ImageJ software. The density values of all specimens stained within the same experiment were then normalized to the group mean (i.e., all sections of all cochlear turns stained in the same experiment gave an average value of 1.0). This correction allowed to compensate for the high inter-trial variation of staining intensity. Within every single experiment, the same number of GC-B WT and GC-B KO sections were stained by the same experimenter. These sections were in parallel exposed to the identical solutions, antibody concentration, temperature, and environmental variations. All sections from one mouse were then averaged and entered the statistical evaluation as *n* = 1.

### X-gal Staining

For X-gal staining, brain slices and isolated cochleae, slit from the apex to base, were incubated with the β-galactosidase staining solution containing 0.5 mg/ml X-gal (Sigma) as described (Chumak et al., 2016). Whole-mount X-gal staining of mouse embryos was performed as described previously (Ter-Avetisyan et al., 2014). In brief, embryos were fixed, their tissues equilibrated and stained in β-gal wash solution containing 0.5 mg/ml X-gal and 5 mM potassium ferrocyanide and ferricyanide. After development of a blue color, the reaction was stopped and after washing in PBS, the probes were post-fixed in 4% paraformaldehyde (PFA) and further processed for clearing before microscopic analysis. For cochlear staining of adult mice, isolated cochleae, slit from apex to base, were incubated with the β-gal staining solution containing 0.5 mg/ml X-gal (Sigma), 5 mM K_3_[Fe(CN)_6_], and 5 mM K_4_[Fe(CN)_6_] in PBS complemented with 20 mM MgCl_2_, 0.01% sodium deoxycholate, and 0.02% Nonidet-P40 overnight at 37°C. After incubation, the cochleae were decalcified, embedded, and cryosectioned as described (Zuccotti et al., 2012). The presence of β-gal protein was visualized with the enzyme’s substrate (X-gal), resulting in a blue precipitate only in cells which express the inserted lacZ reporter cassette in exon 1 of the *GC-B* gene.

### Middle Ear Preparation

For middle ear preparations, temporal bones of six mice (three GC-B HET and three KO) with attached bullae were removed from the skull, fixed with 4% PFA for at least 2 h, carefully cleared from soft tissue (glands, muscles, nerves) and then prepared under visual control of a Leica MZ FL III stereomicroscope (Leica, Wetzlar, Germany) mounted with a camera (Soft Imaging System CC12, Olympus) via a microscope objective/camera adapter (0.63x 10446261, Leica). Photographs were taken with CellSens software (version 1.8.1, Olympus Software Imaging Solutions, Olympus).

### Statistical Analysis

Unless otherwise stated, all data were presented as group mean ± standard deviation (SD) or ± SEM. Differences of the means were compared for statistical significance either by Student’s *t*-test, ANCOVA, one-way, or two-way ANOVA and *post hoc t*-test (with α level Bonferroni-adjusted for multiple testing). For statistical evaluation of integrated density from immunohistochemical stainings, the two-sided Student’s *t*-test for independent samples was used. Statistical significance was tested at α = 0.05 and resulting *p*-values are reported in the figure legends. (^∗^): *p* < 0.1; ^∗^: *p* < 0.05; ^∗∗^: *p* < 0.01; ^∗∗∗^: *p* < 0.001; n.s., not significant.

## Results

### GC-B Is Expressed in Sensory Neurons of the Inner Ear

**FIGURE 1:**
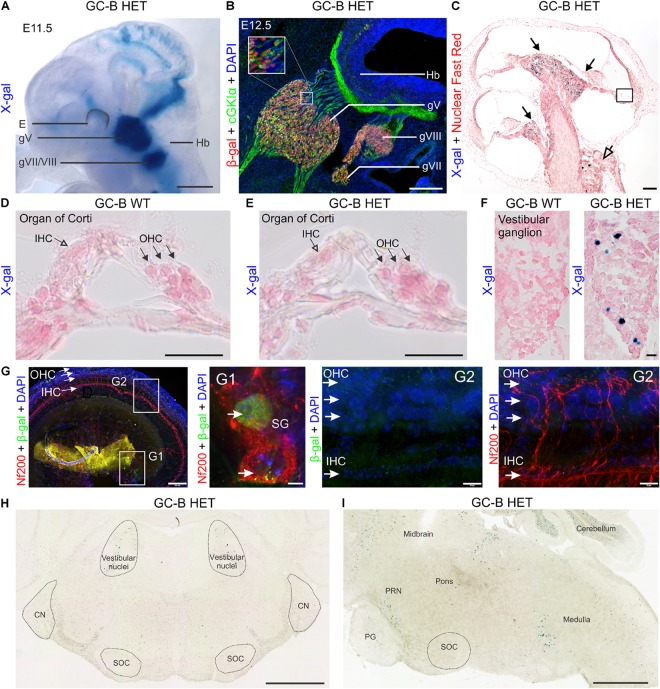
Expression mapping of GC-B in the inner ear by X-gal staining using the *Npr2-lacZ* reporter and by immunofluorescent detection of β-gal. **(A)** β-Gal activity in *Npr2^lacZ^*^/+^ reveals sites of GC-B expression. Due to the presence of a nuclear localization signal, β-gal activity in the *Npr2-lacZ* reporter line is restricted to the nuclei of GC-B expressing cells. X-gal staining in *Npr2^lacZ^*^/+^ whole-mount embryo preparations at embryonic day (E) E11.5 shows a strong expression of GC-B in all cranial sensory ganglia (CSGs) (Ter-Avetisyan et al., 2014), including the trigeminal (gV) and acousticofacial ganglion (gVII/VIII). **(B)** Immunofluorescent detection of β-gal, co-immunostained with cGKIα, in parasagittal sections of the hindbrain region from E12.5 *Npr2^lacZ^*^/+^ embryos shows an overlapping distribution of β-gal with cGKIα expression in the CSGs at the level of CSG gV (trigeminal), gVII (facial), gVIII (vestibuloacoustic) and cGKIα expression in cranial sensory axons. **(C)** Cochlear section of a 10 week-old GC-B HET mouse stained for X-gal (blue) and Nuclear Fast Red (red) with the apex oriented upwards. X-gal-positive cells in GC-B HET mice are indicated by filled (SGNs) or open (vestibular ganglion neurons) arrows. **(D,E)** No X-gal staining was found in the organ of Corti, neither in inner hair cells (IHCs) nor outer hair cells (OHCs) (**E** shows a magnification from boxed areas in **C**). IHCs are indicated by open arrowheads and OHCs by filled arrowheads. **(F)** X-gal staining of sections from vestibular ganglia show a number of X-gal positive nuclei in the inner ear of GC-B HET mice (right panel). **(G)** Whole-mount preparations of an adult GC-B HET mouse immunohistochemically stained with anti-Nf200 (red) and anti-β-gal (green). **(G1)** and **(G2)** show a magnification of boxed areas for spiral ganglion neurons (SG) and organ of Corti, respectively. **(H)** Frontal and **(I)** sagittal sections of a P15 mouse brain show no X-gal staining in auditory brainstem regions (SOC, NC). Positive X-gal staining appears in the regions of vestibular ganglia and in regions related to the pontine reticular nuclei (PRN), close to the pontine gray (PG), and in the hindbrain (medulla). E, eye; g, ganglion; Hb, hindbrain. Nuclei in **(B**,**G)** were stained with 4′,6-diamidin-2-phenylindol (DAPI; blue). Scale bars: 1 mm **(A)**; 25 μm **(B)**; 100 μm **(C)**; 25 μm **(D–F)**; 100 μm (**G** left); 10 μm **(G1,G2)**; 1 mm **(H,I)**.

The GC-B expression pattern in the inner ear and its associated vestibuloacoustic ganglion was analyzed in embryonal (E11.5 and E12.5) and adult (10 weeks-old) *Npr2^lacZ/+^* (GC-B HET) mice. In embryonic whole mounts we used X-gal staining of GC-B HET mice. At E11.5, GC-B is expressed in the acousticofacial ganglion (gVII/VIII, **Figure 1A**) that contains the somata of the neurons which innervate the auditory (organ of Corti) and vestibular (labyrinth with cristae ampullaris in semicircular canals and otolithic organs utricle and saccule) sensory hair cells (Whitfield, 2015). When we used immunostaining in parasagittal sections of E12.5 embryos to co-label β-gal (representing GC-B detected in the nuclei of cells) with cGKIα expression (detected in the cytosol) we found overlapping expression patterns of GC-B with cGKIα in the vestibuloacoustic ganglion (gVIII) and cGKIα expression in its sensory axons (**Figure 1B**) (Ter-Avetisyan et al., 2014). Also in the adult cochlea, in line with DRG neurons at postnatal day (P) 75 (Tröster et al., 2018), a small number of X-gal positive cells were found in SGNs and vestibular ganglion neurons from GC-B HET mice (**Figure 1C**).

No X-gal staining was found in the organ of Corti, neither in IHCs nor OHCs. This is shown through the absence of X-gal staining in cryosections of adult cochleae of GC-B HET in comparison to GC-B WT (**Figures 1D,E**) while GC-B HET exhibited positive X-gal staining in vestibular neurons (**Figure 1F**, right panel). To strengthen the absence of X-gal staining in hair cells we moreover performed whole-mount preparation of cochleae from adult GC-B HET mice (**Figure 1G**), that did show positive anti-β-gal staining in SGNs (**Figures 1G,G1**, middle panel), while no anti- β-Gal staining was seen at the level of hair cells, neither in IHCs nor in OHCs (**Figure 1G**, right panels). To get more insight into the expression pattern of GC-B in target neurons of the AN we moreover performed X-gal staining in sections of the brain of GC-B HET mice as described in Ter-Avetisyan et al. (2014). As shown in **Figure 1H** (left panel) in coronal sections of GC-B HET at P15 for Bregma -5.34 X-gal staining is absent in regions of the CN and superior olivary complex [SOC, including MOC and lateral olivocochlear bundle (LOC)] while positive X-gal staining was restricted to small regions at the level of the vestibular nuclei. This is confirmed when sagittal sections of P15 GC-B HET animals were visualized at the interaural-lateral 1.20 level (**Figure 1I**). While no X-gal staining is seen at the level of the SOC (MOC or LOC), positive staining could be here observed again in the region of the vestibular nuclei and in a region we may assign the region of the pontine reticular nuclei (PRN). From the undetectable expression of GC-B in the cochlea as well as in target regions of the AN in the central auditory brainstem areas, we may conclude that any possible phenotype of GC-B deletion is unlikely linked to GC-B functions in hair cells and axon branching in central auditory neurons.

As only homozygous, but not heterozygous, loss of *GC-B* function leads to a bifurcation deficit during early embryonal stages (Ter-Avetisyan et al., 2014), we hypothesized that any identified functional phenotype in GC-B KO mice (bifurcation deficit) should be absent in GC-B HET (no bifurcation deficit) mice. GC-B HET mice should instead respond indistinguishable from GC-B WT mice.

### GC-B KO Mice Show Moderately Elevated Hearing Thresholds Despite Normal Inner and Outer Hair Cell Phenotype

**FIGURE 2:**
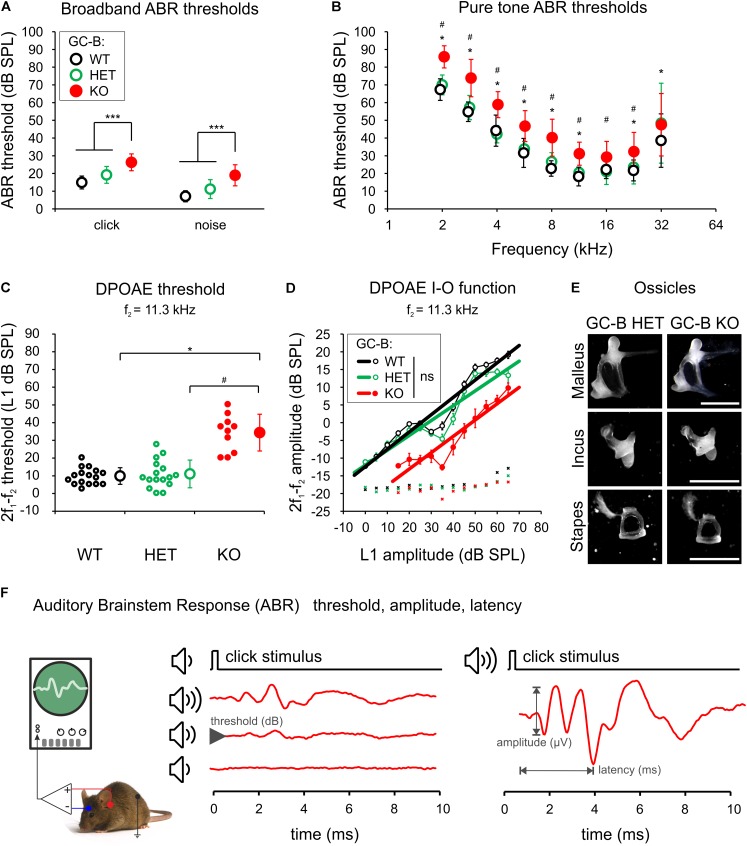
Mild elevation of auditory brainstem response (ABR) and distortion product otoacoustic emission (DPOAE) thresholds in GC-B KO mice but no indication of a conductive hearing loss. **(A)** Mean ± SD click- and noise-evoked ABR thresholds were significantly different for GC-B KO (red circle) compared to GC-B WT (black open circle) and GC-B HET (green open circle) mice. **(B)** Pure tone-evoked ABR thresholds significantly differed for GC-B KO and GC-B WT mice (2 – 11.31 and 22.6 – 32 kHz, asterisks) and for GC-B KO and GC-B HET mice (2 – 22.6 kHz, hash signs). **(C)** Mean ± SD cubic (2f_1_–f_2_) DPOAE threshold (open circles with error bars) at the stimulus frequency f_2_ = 11.3 kHz of GC-B KO mice was significantly elevated compared to GC-B WT and GC-B HET mice (small circles: thresholds of individual mice). **(D)** Mean ± SD cubic DPOAE input-output (I-O) functions (circles connected by a thin line) for f_2_ = 11.3 kHz of GC-B WT, GC-B HET, and GC-B KO mice. The DPOAE threshold difference in **(C)** was reflected by a right-shift of the I–O curve for the GC-B KO mice. No significant difference was found for the linear fit of I–O functions of either genotype (GC-B WT: y = 0.4883x – 12.399, *R*^2^ = 0.9363, GC-B HET: y = 0.4191x – 11.986, *R*^2^ = 0.8851, GC-B KO: y = 0.4607x – 22.291, *R*^2^ = 0.8818, bold lines), indicating no damping effect due to conductive problems in the middle ear. **(E)** Ossicles of GC-B KO mice did not show obvious malformation or size differences. **(F)** Scheme of ABR measurement. Scale bars: 1000 μm. The legend keys in **(A)** apply for **(A–C)**.

First, we estimated basic hearing function of WT mice, heterozygous and homozygous *GC-B* mutants, using ABRs (GC-B WT: *n* = 16; GC-B HET: *n* = 17; GC-B KO: *n* = 18 mice) and DPOAEs (GC-B WT: *n* = 16; GC-B HET: *n* = 16; GC-B KO: *n* = 10 mice) in adult mice. We stimulated the auditory system along the entire frequency-hearing range with acoustic broadband stimuli and pure tone stimuli. The click stimuli produce high SPLs primarily at the lower frequencies of the hearing range of a mouse. Noise bursts produce high SPLs also in high frequencies of the hearing range, and pure tone stimuli allow for the frequency specific allocation of auditory responses to a tonotopic place in the cochlea. GC-B KO mice showed a mild ABR threshold elevation that was significant when compared to GC-B WT and GC-B HET mice for click-, noise burst-, and pure tone-evoked ABR thresholds mainly in the low to middle frequency range (**Figure 2A**, left, click: threshold loss of 11.5 and 6.9 dB when compared to GC-B WT and GC-B HET, respectively, one-way ANOVA, *p* < 0.0001, *F* = 20.19, Bonferroni’s multiple comparisons test *p* < 0.0001 and *p* = 0.0007, **Figure 2A**, right, noise: threshold loss of 11.1 and 8.2 dB when compared to GC-B WT and GC-B HET, respectively, one-way ANOVA, *p* < 0.0001, *F* = 24.76, Bonferroni’s multiple comparisons test *p* < 0.0001 and *p* < 0.0001, and **Figure 2B**, pure tones: threshold loss of on average 13.3 and 11.7 dB when compared to GC-B WT and GC-B HET, respectively, see **Supplementary Table 1**). In contrast, ABR thresholds of GC-B WT and GC-B HET mice did not differ significantly for click- (Bonferroni’s multiple comparisons test, *p* = 0.0721), noise burst- (Bonferroni’s multiple comparisons test, *p* = 0.0741), and pure tone-evoked ABR thresholds for the frequency range between 2 and 22.6 kHz (Bonferroni’s multiple comparisons test, see **Supplementary Table 1**). ABR threshold differences between GC-B WT and GC-B HET mice reached statistical significance only at 32 kHz, a test frequency for which measurement variances were generally larger for all genotypes. We therefore focused the interpretation of differences in hearing of GC-B WT, GC-B HET, and GC-B KO on the low to middle frequency range. Otoacoustic emissions were measured as an objective indicator of active cochlear amplification through electromechanical properties of OHCs (Brownell, 1990; Avan et al., 2013). DPOAE thresholds were significantly elevated for stimulation frequency of f_2_ = 11.3 kHz in GC-B KO (one-way ANOVA, *p* < 0.0001, *F* = 38.42) compared to GC-B WT (Bonferroni corrected *p-*value, *p* < 0.0001, GC-B WT: *n* = 16, GC-B KO: *n* = 10) and GC-B HET mice (**Figure 2C**, Bonferroni corrected *p-*value, *p* < 0.0001, GC-B HET: *n* = 16). This implies that the ABR threshold elevation in the middle frequency range at 11.3 kHz (**Figures 2A,B**) arises in part from deficits in OHC function in GC-B KO mice.

A loss of GC-B function impairs endochondral ossification, resulting in dwarfism (Tsuji and Kunieda, 2005). To exclude that the DPOAE responses and threshold differences were confounded by malformations of middle ear bones (ossicles), we inspected the sound conductance of the middle ear ossicular chain and the morphology of the middle ear and the ossicles themselves. Slopes of DPOAE I-O functions change with abnormal sound conductance (Turcanu et al., 2009; Qin et al., 2010). For emission signals evoked at f_2_ = 11.3 kHz stimulus frequency, linear regression analysis did not reveal a difference in DPOAE I–O function slopes between GC-B KO and GC-B WT [**Figure 2D**, ANCOVA, *p* = 0.6813, *F*(1,21) = 0.173] or GC-B KO and GC-B HET mice [**Figure 2D**, *p* = 0.5755, *F*(1,21) = 0.323]. No size differences or obvious malformations of the ossicles were observed in [**Figure 2E**, representative for GC-B HET: *n* = 3, GC-B KO: *n* = 3 mice]. Also the auditory bulla of GC-B KO mice was inconspicuous in size and shape (not shown). We can, however, not exclude that head size and shape differences as well as differences in body size, weight, and bone stiffness may interfere with acoustical and electrophysiological measurements, reflected particularly in a larger ABR wave I size.

**FIGURE 3:**
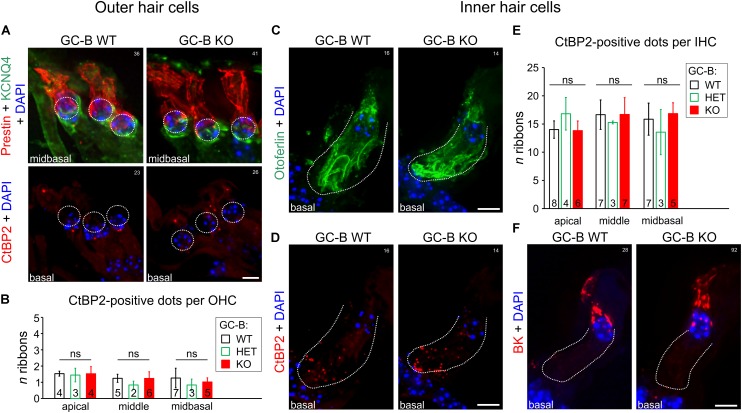
No change in inner hair cell (IHC) or outer hair cell (OHC) phenotype or reduction in numbers of afferent synaptic contacts with OHCs or IHCs was observed in GC-B KO mice. **(A)** Representative immunohistochemical staining of OHCs of GC-B WT (left panels) and GC-B KO (right panels) mice with antibodies targeted against KCNQ4 (green; upper panels) and prestin (red; upper panels) shown for midbasal cochlear turns. GC-B WT and GC-B KO OHCs did not differ in OHC marker protein expression. No differences for the OHC synaptic ribbons (red; CtBP2; lower panels) was observed. **(B)** Quantification of OHC ribbons in apical, middle, and midbasal cochlear turns. Number of OHC synaptic ribbons did not significantly vary between genotypes in the three studied cochlear turns. Bars in **(B)** represent mean ± SD ribbons per OHC; numbers in bars indicate the *n* of mice studied. Representative immunohistochemical staining of IHCs of GC-B WT (left panels) and GC-B KO mice (right panels) with antibodies against otoferlin (**C**, green), CtBP2/RIBEYE (**D**, red), and BK (**F**, red) shown for midbasal cochlear turns. Overall otoferlin staining, number of IHC synaptic ribbons (CtBP2), and BK expression were not different between genotypes. **(E)** Quantification of IHC ribbons in cochlear divisions of apical, middle, and midbasal turns representing low, middle and high frequency coding IHCs, respectively. No significant difference in number of synaptic ribbons was found between genotypes for either cochlear turn. Bars in **(E)** represent mean ± SD ribbons per IHC, numbers in open bars indicate the *n* of mice from which the IHC ribbons were counted from. Nuclei in **(A,C,D,F)** were stained with 4′,6-diamidin-2-phenylindol (DAPI; blue). Scale bars in **(A,C,D,F)**: 5 μm. White numbers 14–41 indicate experiment numbers.

The threshold difference detected in GC-B KO mice might be due to an impaired final differentiation of OHCs. The final differentiation of OHCs can be judged at a first sight on the basis of the characteristic distribution of KCNQ4 in the basal part of the OHC membrane (Rüttiger et al., 2004; Mustapha et al., 2009), where KCNQ4 proteins carry the potassium current *I*_K,n_, from hearing onset onwards (Marcotti and Kros, 1999). Loss of KCNQ4 not only leads to progressive hearing loss (Beisel et al., 2000; Jentsch et al., 2000; Leitner et al., 2012) but *I*_K__,__n_ currents, a K^+^ current carried by KCNQ4, is most critical for of normal maturation of OHCs as, e.g., the onset of electromotility (Marcotti and Kros, 1999). Also the KCNQ4 redistribution from the overall OHC membrane to the basal part of OHCs coincides with a redistribution of prestin, the protein responsible for cochlear amplification (Dallos, 2008). A mature OHC thus can be recognized through KCNQ4 in the basal pole and prestin with a restricted pattern in the lateral wall (Weber et al., 2002). Exemplarily shown for midbasal turns, the KCNQ4 (green) and prestin (red) expression pattern mirrored an adult-like distribution in the basal and lateral OHC membrane for both the GC-B WT and GC-B KO mice (**Figure 3A**, upper panels). Also, CtBP2/RIBEYE-positive particles in ribbon synapses, essential for maintaining a residential vesicle pool (Becker et al., 2018), afferent fiber activity and timing at stimulus onset (Sheets et al., 2017) are in OHCs typically localized at the basal pole, opposing afferent type II fibers in OHC synapses (Martinez-Monedero et al., 2016). A typical distribution of 1–2 CtBP2/RIBEYE-positive dots at the base of OHCs was indistinguishable between genotypes (**Figure 3A**, lower panels), confirmed by quantification of CtBP2/RIBEYE-positive dots which revealed no significant difference between genotypes [**Figure 3B**; two-way ANOVA, *p* = 0.1864, *F*(2,30) = 1.778, GC-B WT: *n* = 4–7, GC-B HET: *n* = 2–3, GC-B KO *n* = 4–6]. In addition to OHC mechanoelectrical properties, an altered response pattern of IHCs can contribute to the elevated hearing thresholds in GC-B KO mice as well. To examine possible changes in IHC development, we investigated the distribution of otoferlin, the multivalent Ca^2+^-sensitive scaffold protein of IHCs (Hams et al., 2017) that delineates IHC membranes (Duncker et al., 2013). Otoferlin labeling was combined with the ribbon synapse marker CtBP2/RIBEYE, which is lost in conjunction with impaired IHC synapse vesicle release properties and deafferentation (Kujawa and Liberman, 2009). As shown in midbasal cochlear turns, the expression pattern of otoferlin (**Figure 3C**) and CtBP2/RIBEYE (**Figure 3D**) was similar between GC-B WT and GC-B KO mice. Quantification did not reveal significant differences in the number of CtBP2/RIBEYE-positive dots in IHC synapses (**Figure 3E**, two-way ANOVA, *p* = 0.8173, *F*(2,41) = 0.2027) between genotypes. Further, GC-B KO mice maintained normal expression of BK channels in IHCs (**Figure 3F**). Upregulation of BK channels with hearing onset is essential to transform immature IHCs (producing spontaneous Ca^2+^-spikes) into mature functional cells, that are then able to respond to sound stimuli with graded receptor potentials (Marcotti, 2012). Given that the differentiation of OHCs and IHCs was normal in GC-B KO mice, it is highly unlikely that the observed elevated hearing thresholds in GC-B KO mice result from hair cell development failures.

We conclude that an elevation of hearing thresholds observed in GC-B KO mice when compared to GC-B WT mice is unlikely linked to achondroplastic bone growth deficit or a failure of final IHC and OHC differentiation.

### MOC-Efferent Control of OHC Activity Is Reduced in GC-B KO Mice

**FIGURE 4:**
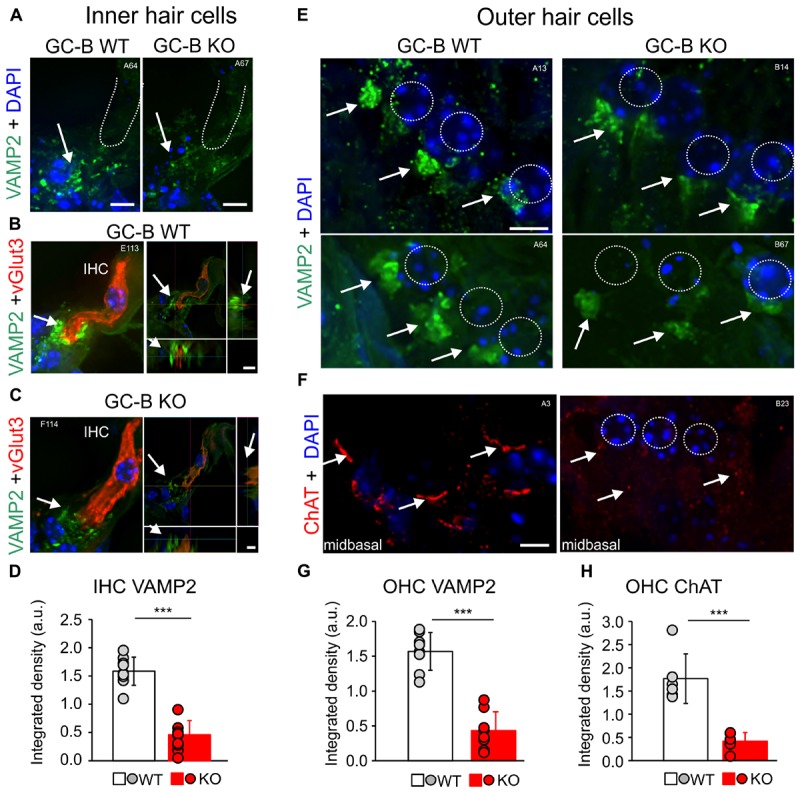
Reduction of efferent innervation of the outer hair cells (OHCs) and inner hair cells (IHCs) in GC-B KO mice. **(A,B,C,E)** Representative immunohistochemical staining with antibodies against Synaptobrevin (vesicle-associated membrane protein isotype 2; VAMP2; green), a marker for membrane bound proteins found in synaptic vesicles of efferent synaptic contacts. **(A)** VAMP2 was reduced in efferent synaptic contacts with IHCs in GC-B KO compared to GC-B WT mice shown for *n* = 2 mice (quantification in **D**, *n* = 9; a.u., arbitrary units). **(B,C)** IHCs were co-stained with the vesicular glutamate transporter 3 (vGlut3, red) to show VAMP2 outside of IHCs. **(E)** VAMP2 expression is reduced below OHCs in GC-B KO shown for *n* = 2 mice (quantification in **G**, *n* = 8). **(F)** Neuronal dendritic processes of cholinergic cochlear efferents near the basal pole of OHCs were stained with antibodies against choline acetyltransferase (ChAT; red). ChAT staining near the basal pole of OHCs was greatly reduced in GC-B KO mice (quantification in **H**, *n* = 6). Nuclei were stained with 4′,6-diamidin-2-phenylindol (DAPI; blue). Scale bars: 5 μm. ^∗∗∗^*p* < 0.001, two-sided Student’s *t*-test. White numbers A13-F114 indicate experiment numbers.

As changes in efferent feedback to hair cells can alter hearing thresholds (Darrow et al., 2007; Maison et al., 2009; Jäger and Kössl, 2016), we next questioned whether the worsened hearing threshold in GC-B KO mice may be linked to an altered efferent feedback control of hair cells. Cholinergic inhibition of cochlear hair cells via olivocochlear (OC)-efferent feedback is mediated by Ca^2+^ entry through α9-/α10-nicotinic receptors, that through Ca^2+^-activated K^+^ channels are accounted for improving the ability to detect signals in noise (Huang and May, 1996; Hienz et al., 1998; Lustig, 2006). Efferent contacts on hair cells are also suggested to protect against noise-induced cochlear injury (Maison et al., 2013a,b) and the cholinergic nerve terminals are involved in regulation of hair cell outward K^+^ currents in a manner dependent on acetylcholine release machinery (Housley et al., 1992; Vetter et al., 2007; Fuchs et al., 2014). We used the efferent marker choline acetyltransferase [ChAT, staining cholinergic fibers (Kitanishi et al., 2013)] and Synaptobrevin (VAMP2, staining membrane bound proteins found in synaptic vesicles) to analyze efferent innervation of the cochlea in greater detail. We investigated cholinergic contacts with immunostaining of cryosections with antibodies against ChAT and VAMP2 in image stacks using high-resolution fluorescence deconvolution microscopy. In GC-B WT mice VAMP2 staining below IHCs was significantly reduced (**Figure 4D**, *n* = 9, *p* < 0.001) as shown exemplarily for a midbasal cochlear turn (**Figure 4A**) and three additional GC-B WT and GC-B KO mice (**Supplementary Figures 1A,B**). Co-staining with the vesicular glutamate transporter 3 (vGlut3), the protein that is essential for hearing (Ruel et al., 2008; Seal et al., 2008) and changes in expression upon IHC disturbance (Lee et al., 2015; Yu et al., 2016) revealed no difference in vGlut3 staining between GC-B WT and GC-B KO mice, while non-overlapping VAMP2 as shown at two different section levels of the IHCs was strongly reduced below IHCs of GC-B KO mice (**Figures 4B,C**). Also on the OHC level VAMP2 was significantly reduced as shown exemplarily for two different GC-B WT and GC-B KO mice (**Figure 4E**) and quantified across the cochlea (**Figure 4G**, *n* = 8, *p* < 0.001). In GC-B KO mice the ChAT staining at the OHC level was also profoundly reduced, exemplarily shown for the midbasal turn (**Figure 4F**) and additional examples (**Supplementary Figures 1C,D**) and quantified across the cochlea (**Figure 4H**, *n* = 6, *p* < 0.001).

**FIGURE 5:**
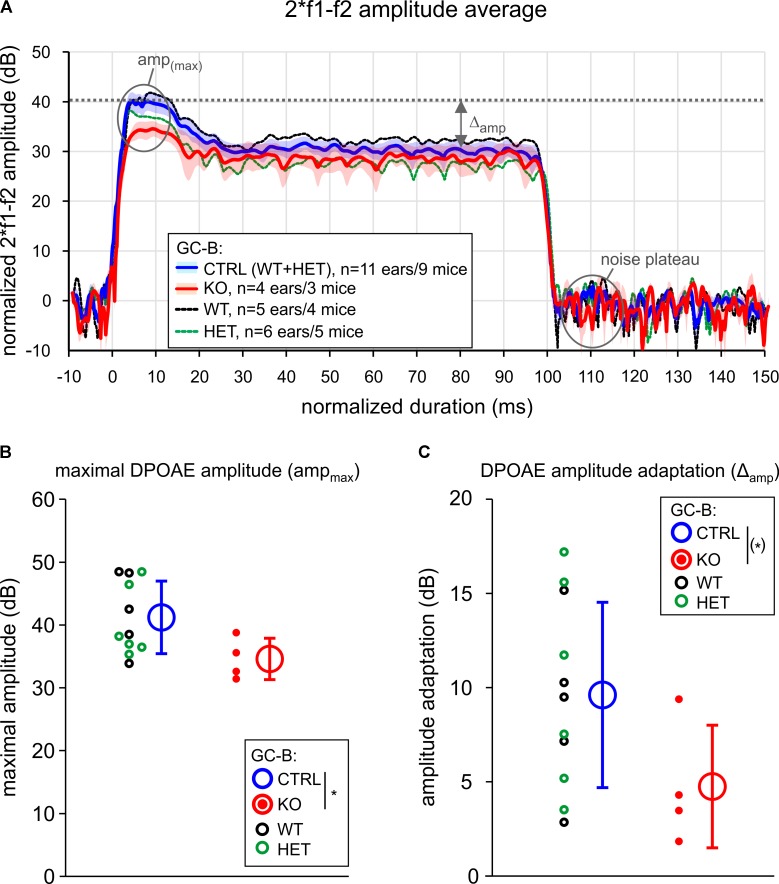
Fast adaptation of distortion product otoacoustic emission (DPOAE) amplitudes was smaller in GC-B KO mice. **(A)** Time course of the 2^∗^ f_1_ – f_2_ DPOAE amplitude signal over 100 ms stimulation (f_2_ = 11.3 kHz, L1 = 70 dB) for groups of GC-B WT, HET, and KO mice. The DPOAE amplitude was normalized to noise level (noise plateau = 0 dB). The maximal DPOAE amplitude (amp_max_) after primary onset, and amplitude adaptation (Δ_amp_) are indicated. No significant difference for Δ_amp_ and amp_max_ was found for GC-B WT (black) and GC-B HET (green) mice. The groups were therefore combined and served as control group (GC-B CTRL, blue line and shade, mean ± SEM) for comparison to GC-B KO mice (red line and shade, mean ± SEM). **(B)** Mean ± SD amp_max_ (open circles and error bars) and **(C)** mean ± SD Δ_amp_ (open circles and error bars) were different in GC-B KO mice (red filled dots: individual ears) when compared to GC-B CTRL mice (black filled dots: individual ears of GC-B WT ears, green filled dots: GC-B HET ears) resulting in a statistical trend for reduced Δ_amp_ values (one-sided Student’s *t*-test, *p* = 0.0464, with *p* < 0.05 certified in bootstrap resampling procedures: *p* = 0.043, resampling with *n* = 11 and *n* = 4 for GC-B CTRL and GC-B KO, respectively, 10,000 resamplings, 0.0406 < *p* < 0.0479 in 20 repetitions). *n* of analyzed ears/mice: GC-B WT (5/4), GC-B HET (6/5), and GC-B KO (4/3) mice.

Aiming to investigate if qualitative evidence for altered efferent terminal size is related to functional deficits of MOC efferents in GC-B KO mice, we reconsidered that previous studies observed up to 10 dB difference in hearing thresholds when, e.g., rapid OHC efferent descending innervation, originating from the MOC in the brainstem (Warr and Guinan, 1979), is dysfunctional (Jäger and Kössl, 2016). The fast neuronal output of MOC-efferents, modifying OHC electromotility, was tested by the rapid (∼100 ms), ipsilateral adaptation (Liberman et al., 1996; Kujawa and Liberman, 2001) of DPOAE amplitudes during stimulus presentation (Maison et al., 2012). The difference in DPOAE amplitude before and after stimulation is presumed to reflect the suppression of OHC mechanoelectrical properties in response to the activation of, mainly ipsilateral, MOC-neurons (Liberman et al., 1996). A subset of adult GC-B WT, GC-B HET and GC-B KO mice were tested for ipsilateral adaptation of DPOAE amplitudes (**Figure 5**) using phase-varied primary pairs as elicitors (Whitehead et al., 1996; Dalhoff et al., 2015). Successful reduction of the DPOAE amplitudes, elicited by f_2_ stimulus SPLs ranging from 60 to 70 dB SPL (Kujawa and Liberman, 2001), was observed in four GC-B WT, five GC-B HET, and in three GC-B KO mice (**Figure 5A**). We observed a significantly smaller maximal DPOAE amplitude in the GC-B KO mice (**Figure 5B**, see also **Figure 2D**) and a statistically non-significant trend for smaller amplitude adaptation in GC-B KO mice (**Figure 5C**) compared to GC-B control mice (GC-B WT and HET). While the reduced adaptation strength could be linked to reduced MOC function, we currently cannot exclude an altered middle ear muscle reflex, both expected to change when auditory input is changed (Valero et al., 2016).

Conclusively, this indicates a trend of reduced capacity of MOC-efferents to suppress DPOAE amplitudes (**Figures 5A,C**), indicating reduced efferent strength.

### Increased and Delayed Supra-Threshold ABR Waves in GC-B KO Mice

**FIGURE 6:**
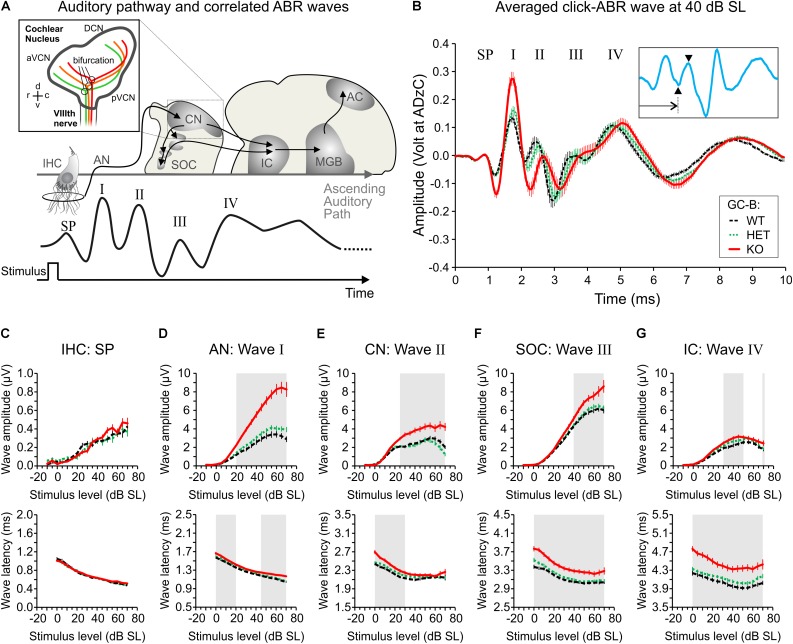
Elevated click-evoked auditory brainstem response (ABR) wave I–IV amplitudes and delayed latencies in GC-B KO mice. **(A)** Schematic drawing of the auditory pathway and correlated stimulus-evoked deflections of ABR waves summating potential (SP), I, II, III, and IV. The insert illustrates axon bifurcation of auditory nerve (AN) fibers from the VIIIth nerve. d, v, r, c refer to anatomical orientation dorsal, ventral, rostral, and caudal, respectively. aVCN, anterior part of the ventral cochlear nucleus; pVCN, posterior part of the ventral cochlear nucleus; DCN, dorsal cochlear nucleus. Colored projections depict fibers from low (green) to high (red) frequency representing tonotopically organized fibers [insert created according to Lu et al. (2014)]. **(B)** Mean ± SEM click-evoked ABR waves recorded at 40 dB SL for GC-B WT (black; *n* = 18), GC-B HET (green; *n* = 20) and GC-B KO (red; *n* = 18) mice. The waves corresponding to the SP, wave I, II, III, and IV are marked. The inset shows a representative GC-B HET mouse ABR waveform (blue line) to illustrate how wave latencies and amplitudes are defined. Wave latencies were defined as the time point of the minimum respective to stimulus onset (arrow) and amplitudes were calculated from the difference between minimum and subsequent maximum of each ABR wave (arrowheads). (**C–G; upper panels**) Mean ± SEM I–O functions of maximum peak-to-peak amplitudes of the SP **(C)** and ABR waves I **(D)**, II **(E)**, III **(F)**, and IV **(G)**. SP amplitude was not significantly different between genotypes **(C)**. Summed neuronal activity of the AN, CN, superior olivary complex (SOC), and inferior colliculus (IC) was significantly elevated in GC-B KO (**D–G, red lines**) compared to GC-B WT (black lines) and of the AN, CN, and SOC to GC-B HET (green lines) mice. Note that ABR wave amplitudes are plotted from –10 to 70 dB SL (normalization to threshold) and that the amplitude scale differs for SP amplitudes. (**C–G; lower panels**) Mean ± SEM I–O functions of ABR wave latencies corresponding to the analyzed peak amplitudes in the upper panels of the corresponding boxes. Wave latency of the SP did not differ between genotypes **(C)**. ABR wave I **(D)**, II **(E)**, III **(F)**, and IV **(G)** latencies were significantly delayed in GC-B KO compared to GC-B WT and GC-B HET mice. Note that latency is increasing along the afferent auditory pathway. Gray-shaded areas in **(D–G)** correspond to stimulus intensities with significant difference between GC-B WT and GC-B KO mice. Statistical comparisons are detailed in (**Supplementary Tables 2**, **3**). The legend keys in **(B)** apply for **(B–G)**.

Medial olivocochlear bundle responses are mainly driven by auditory input. We therefore tested functions of the inner ear and AN in GC-B WT, GC-B HET, and GC-B KO animals. A characteristic property of functional IHCs is the summating potential (SP) generated by the receptor potentials of IHC ensembles (Durrant et al., 1998). The first negativity (deflection) in the ABR waveform fine structure provides an estimate for the SP (**Figure 6A**; SP). The SP can inform on mechanoelectrical transduction (MET) channel currents in IHCs (Tasaki et al., 1954; Patuzzi et al., 1989; Rüttiger et al., 2017). SP wave amplitudes and latencies were analyzed from click-evoked ABRs for increasing SPLs, normalized to their individual ABR thresholds (dB SL). Neither SP amplitudes [**Figure 6C**, upper panel; two-way ANOVA, *p* = 0.3599, *F*(2,1350) = 1.023, GC-B WT: *n* = 19–26 ears, GC-B HET: *n* = 18–31 ears, GC-B KO: *n* = 18–31 ears] nor SP latencies [**Figure 6C**, lower panel; two-way ANOVA, *p* = 0.1266, *F*(2,1241) = 2.070, GC-B WT: *n* = 25–26 ears, GC-B HET: *n* = 30–31 ears, GC-B KO: *n* = 18–31 ears] were significantly different between genotypes. This indicates that the elevation of hearing thresholds in GC-B KO mice is unlikely linked with sound transduction deficits in IHC (MET) channels and confirms a normal differentiation of IHCs in GC-B KO mice as already predicted from findings shown in **Figure 3**.

Fine structure analysis of supra-threshold ABR wave amplitudes was used to define additional hearing loss downstream to OHC damage and loss of threshold sensitivity. Sound-evoked ABR waveform amplitudes change proportionally to the discharge rate and the number of synchronously firing auditory fibers (Johnson and Kiang, 1976). Therefore, a decline in supra-threshold ABR waveform amplitudes informs about synchrony or loss of discharge rates in afferent auditory fibers driven by the IHCs (Rüttiger et al., 2017). ABR peak latencies (absolute latencies) are calculated as the time of occurrence of distinct peaks with reference to stimulus onset (Burkard and Don, 2007).

Auditory brainstem response wave amplitudes were analyzed for latencies corresponding to sound-evoked neuronal activity in the AN (wave I), in the CN (wave II), in the SOC (wave III) and in the lateral lemniscus and inferior colliculus (LL and IC; wave IV) (Melcher and Kiang, 1996) (**Figures 6A,B**; ABR wave). ABR wave amplitudes and latencies were analyzed from click-evoked ABRs for increasing stimulus level (dB SL) in GC-B WT, HET and KO mice. Supra-threshold ABR wave amplitude and latency growth functions were significantly different between GC-B genotypes [GC-B WT: *n* = 18 mice, GC-B HET: *n* = 20 mice, GC-B KO *n* = 18 mice; amplitudes: ABR wave I: two-way ANOVA, *p* < 0.0001, *F*(2,1457) = 354.2; ABR wave II: two-way ANOVA, *p* < 0.0001, *F*(2,1100) = 130.0; ABR wave III: two-way ANOVA, *p* < 0.0001, *F*(2,1460) = 48.31; ABR wave IV: two-way ANOVA, *p* < 0.0001, *F*(2,1447) = 26.34; latencies: ABR wave I: two-way ANOVA, *p* < 0.0001, *F*(2,1340) = 63.20; ABR wave II: two-way ANOVA, *p* < 0.0001, *F*(2,1001) = 48.57; ABR wave III: two-way ANOVA, *p* < 0.0001, *F*(2,1350) = 168.2; ABR wave IV: two-way ANOVA, *p* < 0.0001, *F*(2,1336) = 230.2]. ABR wave I–IV amplitudes for the middle and high stimulus levels (**Figures 6D–G**, upper panels) were significantly increased in GC-B KO compared to GC-B WT mice and ABR wave I–III amplitudes compared to GC-B HET mice (Bonferroni’s multiple comparisons test, see **Supplementary Table 2**). Furthermore, ABR wave I and wave II were delayed in GC-B KO mice already at hearing threshold (**Figures 6D,E**, lower panels) and progressively delayed toward high stimulus levels for ABR wave I. Wave III and wave IV were even more delayed (**Figures 6D–G**, lower panels, Bonferroni’s multiple comparisons test, see **Supplementary Table 3**). In contrast, ABR wave amplitudes or latencies were not found to be different between GC-B HET and GC-B WT mice up to 65 dB SL (Bonferroni’s multiple comparisons test, see **Supplementary Tables 2**, **3**).

In conclusion, IHC differentiation (adult-like expression patterns of marker proteins) and IHC receptor potentials (latency and amplitude of the SP) are similar in GC-B KO and GC-B WT mice. Therefore, the elevated hearing thresholds in GC-B KO mice cannot be linked to obvious deficits in IHC development and function. However, elevated hearing thresholds in combination with elevated early and late ABR waves and delayed late ABR waves in GC-B KO mice may represent functional consequences of reduced MOC-related direct inhibition of OHC function, possibly leading to elevated gain of the cochlea (Guinan, 2006). In parallel to compromised proper efferent feedback control of AN fibers, this may lead to elevated AN thresholds and delayed AN responses (Ruel et al., 2001; Bourien et al., 2014).

### I–O Functions of Amplitude-Modulated Sounds Are Reduced in GC-B KO Mice

**FIGURE 7:**
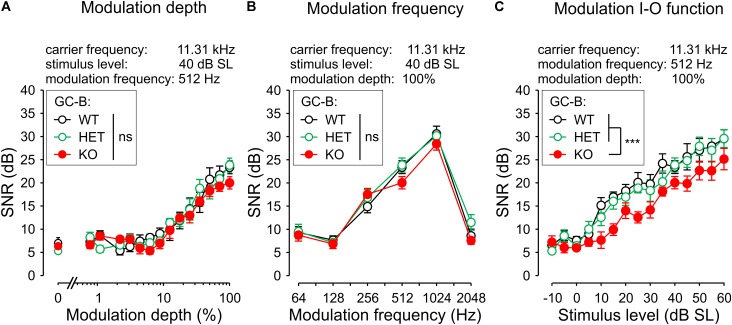
Loss of GC-B results in reduced responses in auditory steady state response input-output (ASSR I–O) function. **(A)** Mean ± SEM signal-to-noise ratio (SNR) modulation depth response (carrier frequency 11.31 kHz; stimulus level 40 dB SL; modulation frequency 512 Hz) was not significantly different between genotypes. **(B)** Mean ± SEM SNR of the response to increasing modulation frequencies (carrier frequency 11.31 kHz; stimulus level 40 dB SL; modulation depth 100%) of GC-B WT (black circles), GC-B HET (green circles) and GC-B KO mice (red circles) did not differ between genotypes. **(C)** Mean ± SEM SNR modulation I–O function of an amplitude-modulated tone (carrier frequency 11.31 kHz; modulation frequency 512 Hz; modulation depth 100%). GC-B KO I–O function was significantly reduced compared to GC-B WT and GC-B HET mice.

To investigate whether the increased and delayed central sound responses (ABR waves II–IV) in GC-B KO mice influence temporal sound processing, we analyzed ASSRs to amplitude-modulated stimuli. ASSRs are used to investigate the integrity of auditory pathways and the cortex by measuring the synchronous, phase-locked discharge of auditory neurons to the modulation frequency of acoustic stimuli (Kuwada et al., 2002). The ASSR is a clear indicator for the proper processing of amplitude-modulated acoustic stimuli in subcortical areas and in the frontocentral cortex (Engelien et al., 2000). In a subset of animals (GC-B WT: *n* = 8; GC-B HET: *n* = 10; GC-B KO: *n* = 8) the ASSR was assessed for a wide range of modulation frequencies, modulation indices (depth), and stimulus levels (I–O function, **Figures 7A–C**). Responses to amplitude-modulated tones with the carrier frequency 11.3 kHz and varying modulation depth (contrasts 0.78–100 and 0%) were not different between genotypes [**Figure 7A**; two-way ANOVA, *p* = 0.1853, *F*(2,345) = 1.694]. Also, the responses to amplitude-modulated stimuli with modulation frequencies ranging from 64 to 2048 Hz at 40 dB SL with 100% modulation depth were not different between genotypes [**Figure 7B**; two-way ANOVA, *p* = 0.1240, *F*(2,138) = 2.119]. This indicates that for stimulus levels at 40 dB SL central auditory circuits in GC-B WT and GC-B KO mice could properly respond to amplitude-modulated stimuli. However, when we tested ASSR I–O functions, evoked by an amplitude-modulated tone of 11.3 kHz and modulated with 512 Hz at 100% modulation depth, a difference between genotypes was observed [**Figure 7C**; two-way ANOVA, *p* < 0.0001, *F*(2,344) = 28.49]. *Post hoc* tests (Bonferroni’s multiple comparisons test, see **Supplementary Table 4**) revealed significantly reduced ASSRs in particular at stimulus levels close to threshold in GC-B KO compared to GC-B WT and GC-B HET mice (at 10 or 15 dB SL, respectively), while GC-B HET were not found to be different at any stimulus level from GC-B WT mice.

In conclusion, significantly reduced ASSR I–O functions in GC-B KO mice for close to threshold stimuli point to reduced capacity to resolve amplitude-modulated sounds, particular at low sound intensities.

### GC-B KO Mice Exhibit Normal Acoustic Startle Response With Altered Temporal Features

**FIGURE 8:**
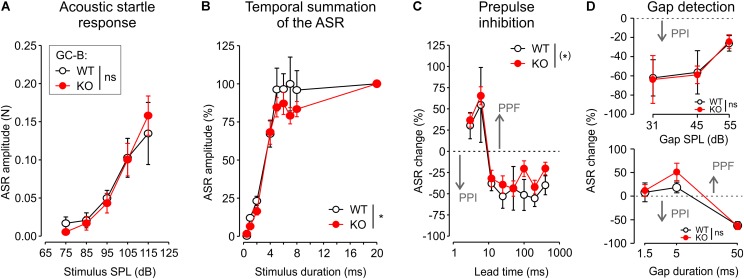
GC-B KO mice maintained subtle changes of temporal features of the acoustic startle response (ASR) and prepulse inhibition (PPI). **(A)** Mean ± SEM ASR amplitude growth of GC-B WT and GC-B KO mice shown for increasing startle stimulus SPL. There was no significant difference between genotypes for stimulus SPLs between 75 and 115 dB SPL. **(B)** In both genotypes ASR amplitude increased with increasing stimulus durations between 0.5 and 20 ms. The mean ± SEM maximum ASR was similar for stimulus durations up to 4 ms in both genotypes but smaller for stimulus durations between 5 and 8 ms in GC-B KO compared to GC-B WT mice. This reduction in temporal summation was significant. **(C)** A prepulse with different lead time was presented before the startle stimulus, which elicited prepulse facilitation of the ASR (PPF, 3–6 ms lead time) or PPI of the ASR PPI (12–400 ms lead time). The broken line marks the zero line for ASR change; values above this line correspond to increase of the ASR (PPF), values below the broken line correspond to reduction of the ASR (PPI). Mean ± SEM change of the ASR resulted in a statistically non-significant trend (*p* = 0.0935) for reduction of PPI in GC-B KO compared to GC-B WT mice. **(D)** Presenting a gap in noise as a startle response modulating prepulse (gap-detection) resulted in similar PPI or PPF in GC-B WT and GC-B KO mice across the explored gap duration (lower panel) and gap SPL (upper panel).

A cluster of afferent auditory neurons, the cochlear root neurons, gives rise to the ASR (Lingenhohl and Friauf, 1994; Gomez-Nieto et al., 2014). The ASR is one of the fastest reflexes found in the mammalian central nervous system (CNS) (Lauer et al., 2017) and a well-established behavioral test in awake animals to test for very fast auditory processing (Steube et al., 2016). To study how impaired AN branching affects proper function of the ASR, acoustic startle behavior was measured in a subgroup of mice (GC-B WT: *n* = 5, GC-B KO: *n* = 7). ASR amplitude increased monotonically with the SPL of the stimulus [two-way ANOVA, *p* < 0.0001, *F*(4,50) = 17.8195] with no observed differences between genotypes [**Figure 8A**; two-way ANOVA, *p* = 0.991, *F*(1,50) = 0.0001; no significant interaction between SPL and genotype: *p* = 0.910, *F*(4,50) = 0.25]. There seemed to be, however, a difference in startle threshold. At 75 dB SPL, 60% of the WT, but only 29% of the KO mice had an individual response significantly differing from spontaneous motor activity (one-sample *t*-tests, *p* < 0.05). At 85 dB SPL all WT, but only 71% of the KO mice had a significant response. ASR amplitudes increased with prolonged startle stimuli from 0.5 to 20 ms. The longer the startle stimulus, the stronger was the ASR [two-way ANOVA, *p* < 0.0001, *F*(7,80) = 57.2]. This increase was similar for stimulus durations of 0.5–4 ms between GC-B KO and GC-B WT mice. The ASR at 20 ms startle stimulus duration shows the saturated, maximal ASR (**Figure 8B**). In GC-B WT mice, saturation of the ASR was reached with the startle stimulus durations of 5 ms. In GC-B KO mice, the saturation of the ASR was not yet reached even at 8 ms. The genotypes differed significantly [**Figure 8B**; two-way ANOVA, *p* = 0.0358, *F*(1,80) = 4.56], meaning that GC-B KO mice need longer minimal startle stimulus durations to reach the maximal ASR. The interaction between stimulus duration and genotype was not significant [two-way ANOVA, *p* = 0.84, *F*(7,80) = 0.49]. When an acoustic prepulse was given 3–6 ms prior to the startle stimulus, the ASR increased (prepulse facilitation), while prepulses presented 12–400 ms prior to the startle stimulus resulted in a decrease of the ASR [**Figure 8C**; PPI, two-way ANOVA, *p* < 0.001, *F*(7,80) = 15.6]. The genotypes showed the trend for a smaller ASR change, that was yet not reaching statistical significance [two-way ANOVA, *p* = 0.0935, *F*(1,80) = 2.88; there was no significant interaction between prepulse lead time and genotype, *p* = 0.98, *F*(7,80) = 0.21]. No significant difference between genotypes could be found when using a gap (1.5–50 ms, lead time 50 ms) in noise (65 dB SPL) to modulate the ASR (**Figure 8D**, upper and lower panel), neither for different gap durations [two-way ANOVA, *p* = 0.319, *F*(1,30) = 1.03] nor for different gap SPL [*p* = 0.931, *F*(1,30) = 0.01].

We conclude that GC-B KO mice displayed a normal ASR with perhaps an increased startle threshold and subtle deficits of temporal integration of startle stimuli.

## Discussion

We here describe that GC-B KO mice with a failure of AN bifurcation exhibit significant deteriorations of auditory fidelity and temporal auditory processing. Deficits include (i) a mild but significant elevation of auditory thresholds, linked with (ii) elevated thresholds for OHC motility, (iii) diminished MOC-efferent-induced suppression of OHC motility, (iv) elevated and delayed ABRs, (v) reduced capacity to temporally follow close to threshold amplitude-modulated stimuli, (vi) behavioral deficits in accuracy of temporal summation of the ASR. The deficits are discussed in the context of bifurcation of ANs being essential for proper adjustment of the efferent gain control of cochlear output.

### GC-B KO Mice Have a Mild Audiometric Hearing Loss Despite Normal Phenotype of OHCs and IHCs

Sensory axons of SGNs, among other CSGs, and DRGs fail to form T-like branches in the hindbrain or the spinal cord, respectively, in the absence of GC-B; therefore, bifurcation of the AN in GC-B KO mice is prohibited (Schmidt et al., 2007, 2009; Schmidt and Rathjen, 2010; Ter-Avetisyan et al., 2014). The expression profile of GC-B in the *Npr2-lacZ* reporter mouse can be visualized via X-gal staining, as a *lacZ* expression cassette has been introduced into exon 1 of the *Npr2* gene (Ter-Avetisyan et al., 2014), here shown for the SGNs in the inner ear at embryonal and mature postnatal stages as well as for sections at the brainstem level, excluding GC-B expression in hair cells or target neurons of the AN. We demonstrate for the first time a mild but significant elevation of hearing thresholds in GC-B KO in comparison to GC-B HET and GC-B WT mice. This finding supports the notion that the remaining WT allele in GC-B HET mice can fully compensate for the loss of one (mutant) *Npr2* allele, due to the fact that bifurcation of the AN is unaffected in GC-B HET mice (Ter-Avetisyan et al., 2014). In a spontaneous loss of function mouse mutant *Npr2^cn/cn^*, which carries a missense point mutation (L885R) in the guanylyl cyclase domain of the *Npr2* gene, normal hearing thresholds have been described (Tsuji and Kunieda, 2005; Lu et al., 2014). It is possible that variations in the sensitivity of the method used to determine hearing thresholds between previous and current studies may have led to masking of these subtle differences. In the present study we preclude that the observed elevated hearing thresholds in GC-B KO mice are caused by ossicle-related sound propagation deficits in the middle ear (conductive hearing loss, Gehr et al., 2004; Turcanu et al., 2009; Qin et al., 2010) and we demonstrate that hearing is affected by elevated DPOAE thresholds. This may first indicate that the integrity of the cochlear amplifier is affected in a way that depends on electromotile activity of OHCs (Dallos, 2008). Since, however, GC-B is not expressed in hair cells (**Figure 1**) and GC-B KO mice maintain prestin expression, the protein responsible for electromotility of OHCs (Dallos, 2008), in the lateral OHC membrane, we assume that basic electromotile properties are normal. This is strengthened demonstrating that KCNQ4, essential for hearing (Kharkovets et al., 2006; Holt et al., 2007), normal OHC maturation and development of mature electromotile responses (Marcotti and Kros, 1999), exhibits normal distribution at the basal pole of the OHC membrane (**Figure 3A**), a phenomenon that only gradually occurs with normal onset of hearing (Weber et al., 2002). While this finding alone does not entirely rule out deficits of OHC electromechanical properties, it nevertheless suggests that the elevated DPOAE thresholds in GC-B KO mice are not primarily linked to perturbed final differentiation of OHCs. Also differentiation of IHCs is unlikely to be impaired in GC-B KO mice and thus unlikely to be linked to the hearing deficits in GC-B KO mice as evidenced by normal SP amplitudes, indicating functional MET currents of IHCs (Durrant et al., 1998; Barral and Martin, 2011). Moreover, IHCs of GC-B KO mice do not differ from GC-B WT mice in their expression pattern of CtBP2/RIBEYE-positive synaptic contacts, otoferlin, BK expression (**Figures 3C–F**) and vGlut3 expression (**Figure 4**). Normal number of CtBP2/Ribeye-positive particles in ribbon synapses, previously shown to be essential for maintaining a residential vesicle pool (Becker et al., 2018), afferent fiber activity and timing at stimulus onset (Sheets et al., 2017) may suggest that the observed retrocochlear changes observed in GC-B KO mice may not be associated to IHC synapse deficits. Also, vGlut3 is essential for hearing (Ruel et al., 2008; Seal et al., 2008) and its protein expression level is highly sensitive for any IHCs disturbance as shown in various studies (Lee et al., 2015; Yu et al., 2016). Thus, the observation that vGlut3 staining between GC-B WT and GC-B KO mice, despite profound differences in the level of, e.g., the efferent marker protein VAMP2 under the same condition (**Figure 4B**), further strengthened that GC-B KO AN activity differences may unlikely be linked to deficits of IHC synapses.

### GC-B KO Mice Show Reduced MOC-Efferent Modification of OHC Activity

The present findings rather indicate that the elevated DPOAE threshold in GC-B KO mice may be a result of disturbed retrocochlear feedback control that modifies OHC amplification properties. This is underscored by (i) smaller than normal VAMP2 or ChAT positive efferent terminals below OHCs and (ii) smaller than normal amplitude of DPOAE suppression following ipsilateral stimuli.

(i) Cholinergic inhibition of cochlear hair cells via OC-efferent feedback is mediated by Ca^2+^ entry through α9-/α10-nicotinic receptors, that through K^+^ channels activated by Ca^2+^ in OHCs, reduce sound-evoked electromotile properties of OHCs (Guinan, 2006). Using markers to identify cholinergic fibers (ChAT) (Kitanishi et al., 2013) and membrane bound proteins found in synaptic vesicles (Synaptobrevin, VAMP2), we observed in high-resolution deconvoluted image stacks of cochlear tissues from GC-B KO mice lower ChAT and VAMP2 fluorescence intensity levels below OHCs, as quantified for VAMP2 and ChAT (**Figure 4** and **Supplementary Figure 1**). Indeed, altered morphology of efferent synapse terminals in response to changes in neuronal activity or lack of post-synaptic receptors as, e.g., nicotinic acetylcholine receptors (Vetter et al., 2007; Fuchs et al., 2014) has been described before. Since enlarged MOC efferent synapse size was linked to facilitated MOC response properties (Wedemeyer and Vattino, 2018), we may hypothesize that vice versa smaller MOC efferent synapses, as here shown for GC-B KO mice, may be linked to reduced MOC responses.

(ii) Reduced DPOAEs amplitudes are shown in the present study in GC-B KO mice, a deficit that was associated with a smaller capacity to rapidly reduce DPOAEs amplitude after ipsilateral stimulation of MOC-efferents (**Figure 5**). Typically, when the MOC system is stimulated at the floor of the IVth ventricle, there is a significant reduction in the amplitude of DPOAEs (Maison et al., 2007), resulting in reduced gain of the cochlea (Guinan, 2006). We here observed that ipsilateral stimulation leads to less pronounced fast suppression of OHC mechanoelectrical properties in GC-B KO mice (**Figure 5**), a feature that points to reduced MOC-efferent neuronal output. An impairment of fast MOC-efferent responses by contralateral cortical microinjections of lidocaine was only recently linked with changes in hearing levels by up to about 10 dB (Jäger and Kössl, 2016), the range of hearing threshold elevation found here in GC-B KO mice. The finding was discussed in the context of an inappropriate noise cancelation by MOC-efferents (Huang and May, 1996; Hienz et al., 1998; Lustig, 2006; Millman and Mattys, 2017). While the question of how the observed reduction in rapid compression of OHC function in GC-B KO mice is related to elevated DPOAE thresholds in GC-B KO mice needs further specification, our findings strongly suggest a link of bifurcation deficits and deficits in centrifugal auditory gain control to OHCs.

### GC-B KO Mice May Show Deficits in LOC-Efferent Modification of AN Activity

In addition to reduced MOC-efferent strength, delayed and elevated ABR waves were observed in GC-B KO mice although IHCs of GC-B KO mice had a normal phenotype. Labeling of otoferlin, BK, and IHC ribbons appeared to be similar in GC-B KO and GC-B WT mice, while their expression patterns have been shown to be disturbed when, e.g., axo-somatic MOC-efferents are not functional (Johnson et al., 2013; Sendin et al., 2014; Knipper et al., 2015). This finding may be unexpected regarding that deletion of the MOC-efferent acetylcholine receptor α9 leads to changed tonotopic organization of SOC auditory circuits (Clause et al., 2014) as does the *Npr2* mutation in GC-B KO mice on the level of the CN (Lu et al., 2014). Future studies are essential to correlate deficits in tonotopic organization with the integrity of axo-somatic MOC-efferent fibers in GC-B KO mice. In contrast, the results of the present study support the hypothesis that the elevated supra-threshold ABR amplitudes of the AN observed in GC-B KO mice are primarily linked with a disturbed LOC-efferent feedback control. LOC-efferent fibers contact SGN dendrites below IHCs after hearing onset, extensively influencing its firing rate properties (Le Prell et al., 2003; Darrow et al., 2007; Elgoyhen and Katz, 2012). Lesion of LOC-efferent fibers enhances mean ABR amplitudes in the ipsilateral ear, an observation that was not attributable to OHC-based effects (Darrow et al., 2007). Thus, the elevated ABR wave amplitudes, observed in GC-B KO mice, may be linked to dysfunctional axo-dendritic control of AN excitability, here demonstrated through reduced VAMP2 positive terminals below IHC synapses (**Figure 4**). In the mature cochlea, axo-dendritic efferent feedback control from the LOC includes dopamine, acetylcholine, GABA, enkephalins, dynorphins, and calcitonin gene-related peptide that can be co-localized within the same LOC neuron (Safieddine and Eybalin, 1992; Safieddine et al., 1997). Pharmacological abolishment of tonic-inhibitory dopaminergic contacts onto AN fibers leads to elevated AN thresholds (Ruel et al., 2001). Also, upon loss of GABA_A_ receptor subunits β2 in AN fibers, hearing thresholds are elevated by a certain amount (Maison et al., 2006). This means that two characteristics of ABR in GC-B KO mice - (i) elevated ABR thresholds and (ii) enhanced supra-threshold ABR amplitudes - would point to a disturbed efferent feedback control on the level of the AN.

### GC-B KO Mice Exhibit Altered Processing in the AN

The dynamic range of hearing is made up by the central auditory processing of information transmitted from auditory fibers with various spontaneous firing rates. Fibers with high spontaneous rates (high-SR fibers) respond to sound stimuli close to hearing threshold while fibers with low spontaneous rates (low- to medium-SR fibers) respond at increasing or high SPLs with a rate-level function proportional to stimulus level, while high-SR fibers are already saturated at these levels. Accordingly, high-SR fibers are more sensitive to low SPL stimuli, whereas low-SR fibers have elevated thresholds by about 20-40 dB (Sachs and Abbas, 1974; Yates, 1991). More importantly, the low-SR, high threshold characteristics are established prior to hearing onset when IHCs still generate spontaneous Ca^2+^-spikes under the influence of transient axo-somatic cholinergic input, while the high-SR, low threshold fiber characteristics develop with delay with or after hearing onset (Grant et al., 2010). From that time onwards, the high-SR fibers contribute with their high discharge rate to the compound action potential threshold of the AN, equivalent to ABR wave I (Bourien et al., 2014), potentially influenced by tonic dopaminergic axo-dendritic efferent feedback (Ruel et al., 2001; Knipper et al., 2015). Moreover, AN single fiber responses in gerbils and guinea pigs after ototoxic exposure indicate that AN fibers with the lowest SRs do not contribute to the compound action potential (Bourien et al., 2014). Conclusively, it is unlikely that the observed elevated ABR wave I amplitudes in GC-B KO mice would result from response property changes of low-SR, high threshold fibers. Therefore, it is self-evident to consider complications for proper adjustment of high-SR, low threshold fibers under conditions of impaired AN fiber bifurcation. Of particular interest is how the AN fiber properties are altered, i.e., the low thresholds and narrow dynamic ranges at the characteristic frequencies (Ohlemiller and Echteler, 1990; el Barbary, 1991; Taberner and Liberman, 2005). Moreover, at any given characteristic frequency place in the auditory system, high-SR fibers have the shortest latencies and low-SR fibers have the longest latencies (Rhode and Smith, 1986). Consequently, deficits in high-SR, low threshold fibers could also best explain the severe latency shifts in late ABR responses in GC-B KO mice.

### GC-B KO Mice Show Reduced Brain Responses to Amplitude-Modulated Stimuli

In the present study, we observed that GC-B KO mice differed from GC-B HET or GC-B WT mice in their sensitivity to respond to amplitude-modulated tones (**Figure 7C**). The significantly reduced ASSR in GC-B KO mice may indicate, that low-SR, high threshold fibers are necessary to exploit the whole dynamic range. Indeed, low-SR, high threshold fiber responses mainly contribute to the ASSR, as they surpass high-SR, low threshold fibers in their synchronization to amplitude-modulated stimuli (Joris et al., 2004). In line, poorer coding of the temporal envelope of sound stimuli has been preferentially linked to deafferentation (synaptopathy) of the high-vulnerable low-SR, high threshold fibers (Liberman et al., 2016; Liberman and Kujawa, 2017). Envelope following responses of the ASSRs are not affected in noise background when low-SR, high threshold fibers are intact (Guest et al., 2017), as envelope following responses in noise critically depend on low-SR fibers (Joris et al., 2004), but when tested in quiet, under conditions where both, low-SR and high-SR auditory fibers contribute to amplitude-modulated coding, ASSRs can also be impaired in case high-SR auditory fiber responses are affected (Paul et al., 2017). GC-B KO mice maintained delayed ABR wave IV latencies (**Figure 6G**, lower panel) in the corresponding upper brainstem region, where the majority of critical modulation-sensitive neurons in the brain are located (Schreiner and Langner, 1988). We therefore suggest that the delayed ABRs we observed in GC-B KO mice are due to undamped auditory fiber activity with lowered temporal envelope resolution at lower stimulation levels, resulting in reliance deficits, reduced sensitivity, and poor coding of temporal envelopes.

### GC-B KO Mice Show Subtle Changes of ASR

The ASR is one of the fastest reflexes found in the mammalian CNS (for review see: Lauer et al., 2017), evolutionary essential for survival, and – by that – genetic modifications with impairments would be aggrieved by evolutionary negative selective pressure. We used behavioral tests for ASR (in awake animals) to test how the observed auditory processing deficits in GC-B KO mice may influence auditory elicited behavioral responses. While GC-B KO mice showed similar ASR amplitudes with increasing stimulus SPL and similar PPI in comparison to GC-B WT mice (**Figures 8A,C**), the loudness threshold for inducing an acoustic startle response was higher in individual GC-B KO mice, and temporal summation of the ASR amplitudes showed a statistically significant effect with slower summation in the GC-B KO mice that required prolonged startle stimuli (**Figure 8B**). From this result we propose that the failure of AN fiber bifurcation results in temporal deficits also influencing this fast auditory reflex in GC-B KO mice though we cannot exclude that integration of sound levels fails already at the level of the input side of the ASR, the dendritic input to cochlear root neurons (see, e.g., Merchan et al., 1988). GC-B KO mice displayed a normal strength of ASR, what signifies a rather intact motor component of the ASR. However, due to a shift of the stimulus duration required to reach the maximal ASR amplitudes we may assume that the impaired AN fiber bifurcation led to a reduced input to integrating neurons, thus resulting in a subtle inefficiency of temporal integration. This may impede the ability to quickly respond to very short startle sounds with an applicable ASR. The number of animals was small (WT: *n* = 5, KO: *n* = 7). Nevertheless, already this number clearly showed that sensory-motor integration in KO mice is functional. Since the effect of temporal integration was already significant in these animals, there is no reason in the light of the 3R to increase their number. The startle threshold seemed to be increased in KO mice, since at the lowest startle stimulus SPLs (75 and 85 dB) the number of responding mice was lowered compared to the WTs. This may relate to the threshold increase measured with ABR and DPOAE methods. Again, since the statistical results in the latter measures were very clear, there seemed still no reason to increase the number of animals used.

To conclude the present study, we observed profound auditory processing deficits in GC-B KO mice that include: (i) elevated ABR and DPOAE thresholds, (ii) reduced rapid MOC-efferent responses at the OHC level, (iii) elevated AN responses, possibly linked to disinhibition of efferent feedback control of AN activity, (iv) delayed ABR waves, that could be explained by weakened LOC-efferent control of AN fibers, (v) reduced sensitivity to respond to amplitude-modulated stimuli, what could be explained by compromised reliance of AN firing rate units, and (vi) deficits in temporal resolution of the fast ASR. Regarding that target neurons of AN do not express GC-B as shown in the present study, the previously shown bifurcation deficits of SGNs (Lu et al., 2014; Ter-Avetisyan et al., 2014) might affect all CN regions, in line with disrupted tonotopic organization of AN fiber terminals observed in all divisions of the CN. The observed effect of GC-B KO on ASSR would suggest diminished input in the DCN, linked rather to ASSR responses (Zhao and Liang, 1996), while ABR wave amplitude response deficits would rather be linked to VCN deficits, considering that the differences in ABR waveform likely reflect differences in VCN output activity toward higher brain areas (Melcher and Kiang, 1996). Regarding that the multipolar cells in the VCN, also shown to exhibit deficits in GC-B KO mice (Lu et al., 2014), have been proposed as the interneurons for the cholinergic efferent feedback to the cochlear OHCs thereby controlling cochlear amplification via the MOC (De Venecia et al., 2005), the diminished input to these cells in response to bifurcation loss, may indeed explain efferent feedback control deficits of hair cells. In fact, planar multipolar neurons in the VCN have been shown to make projections to the ventral nucleus of the trapezoid body where they were observed terminating on MOC neurons (Darrow et al., 2012). Thus, our results outline a “raison d’être” why vertebrate cranial nerve fibers should undergo a T-shaped branching. The bifurcation obviously has consequences for the proper activation of second-order neurons in general, and in particular for the temporal features of auditory information processing. Thereby, proper bifurcation of cranial nerve fibers may be regarded as a vital prerequisite for optimized behavioral response. So far, it is unclear which consequences impaired AN fiber bifurcation have for the death of cochlear neurons and for the therapeutic restoration of hearing using cochlear implants (Wilkerson et al., 2017) as well as for auditory synaptopathy and central processing disorders following noise overexposure (Gröschel et al., 2014; Basta et al., 2017). In humans, temporal coding of auditory information remains an indispensable achievement for good speech perception (Shannon et al., 1995; He et al., 2008).

## Author Contributions

SW, DM, SP, DZ, PP, LR, HS, and MiK performed the experiments. SW, DM, SP, DZ, PE, PP, LR, and HS analyzed the data. LR, MaK, RF, and HS conceptualized the study. LR, MaK, SW, DM, PP, HS, and RF wrote or revised the manuscript.

## Conflict of Interest Statement

The authors declare that the research was conducted in the absence of any commercial or financial relationships that could be construed as a potential conflict of interest.
