# Supplementary material for: GC-B Deficient Mice With Axon Bifurcation Loss Exhibit Compromised Auditory Processing
Source: Front Neural Circuits. 2018 Aug 29;12:65. doi: 10.3389/fncir.2018.00065 (PMC6152484; doi:10.3389/fncir.2018.00065)
Supplement: Supplementary file 1 [file Data_Sheet_1.docx]

Supplementary Material

GC-B Deficient Mice With Axon Bifurcation Loss Exhibit Compromised Auditory Processing

Steffen Wolter**, Dorit Möhrle**, Hannes Schmidt, Sylvia Pfeiffer, Dennis Zelle, Philipp Eckert, Michael Krämer, Robert Feil, Peter K.D. Pilz, Marlies Knipper, Lukas Rüttiger*

*** Correspondence:** Corresponding Author: [Lukas.Ruettiger@uni-tuebingen.de](mailto:Lukas.Ruettiger@uni-tuebingen.de)

** These authors have contributed equally to this work.

# Supplementary Figures and Tables

## Supplementary Figures

| **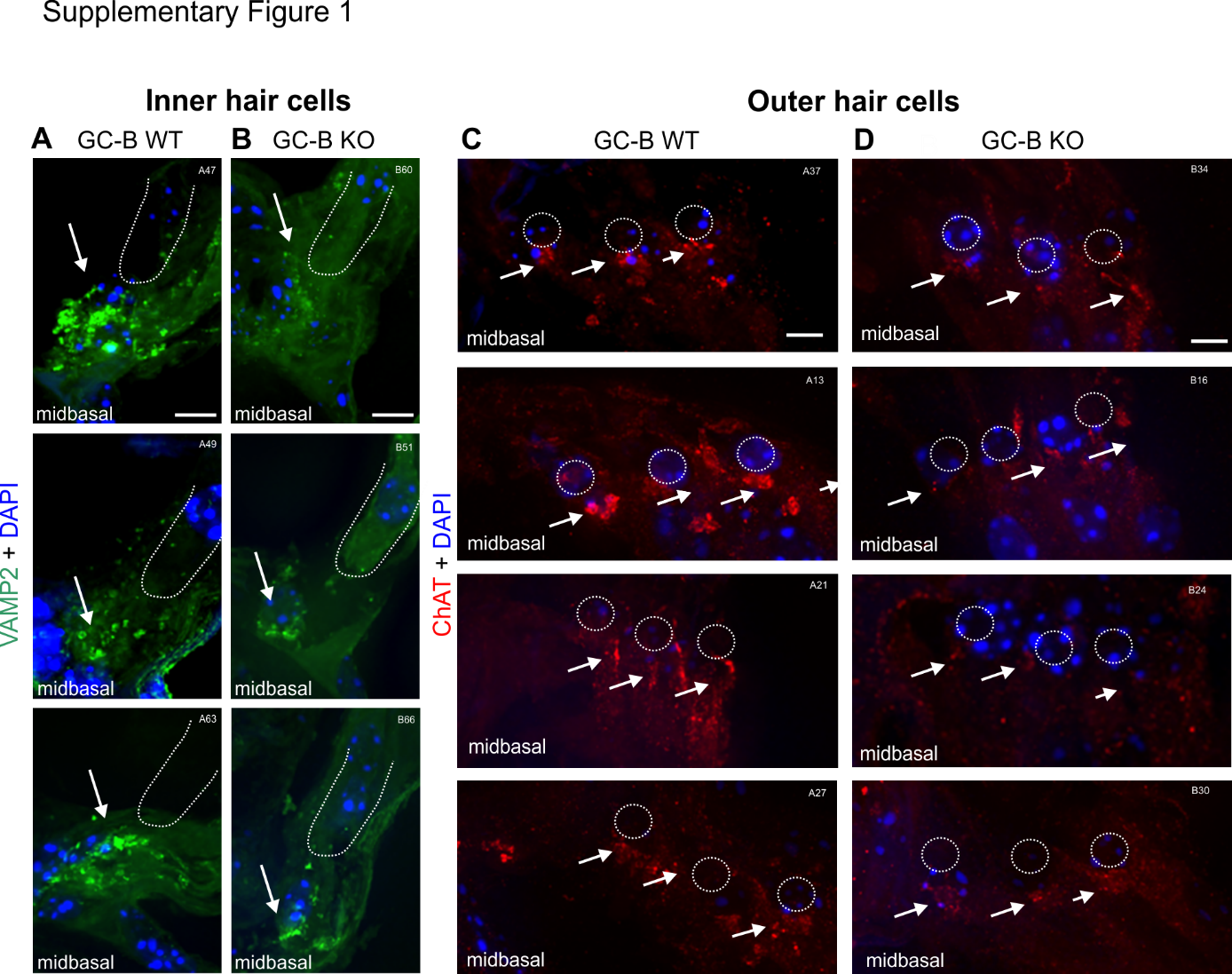** |  |
| --- | --- |

**Supplementary Figure 1.** **Reduction of cholinergic efferent innervation of the OHCs in midbasal cochlear turns in GC-B KO mice.**

**(A,B)** Representative immunohistochemical stainings with an antibody directed against Synaptobrevin (VAMP2; green), a marker for LOC-efferents near the basal pole of IHCs from *n* = 3 GC-B WT **(A)** and *n* = 3 GC-B KO mice **(B)**. VAMP2-positive LOC-efferents below the IHCs were abundant in GC-B WT mice **(A)** but staining showed reduced intensity in GC-B KO mice **(B)**. **(C,D)** Representative immunohistochemical stainings using an antibody specific for choline acetyltransferase (ChAT; red), a marker for cholinergic cochlear efferents at the basal pole of OHCs from *n* = 4 GC-B WT **(C)** and *n* = 4 GC-B KO mice **(D)**. Cholinergic efferent fibers near the basal pole of OHCs were abundant in GC-B WT **(C)** mice but staining was lacking near the basal pole of OHCs in GC-B KO mice **(D)**. Nuclei were stained with 4’,6-diamidin-2-phenylindol (DAPI; blue). Scale bars: 5 µm **(A-D)**. White numbers A13-B66 indicate experiment numbers.

## Supplementary Tables

**Supplementary Table 1:** Statistical comparison of pure tone-evoked ABR thresholds from GC-B WT, GC-B HET and GC-B KO mice.

| **Pure tone ABR thresholds** | | | | | | | | | |
| --- | --- | --- | --- | --- | --- | --- | --- | --- | --- |
| **Frequency**  **(kHz)** | **2** | **2.83** | **4** | **5.66** | **8** | **11.31** | **16** | **22.6** | **32** |
| **WT**  (*n=*14-16)  **vs KO**  (*n=*12-13) | <0.0001  *** | <0.0001  *** | <0.0001  *** | <0.0001  *** | <0.0001  *** | 0.0006  *** | 0.1182  ns | 0.0061  ** | 0.0344  * |
| delta \|dB\| | 18.5 | 19.0 | 14.7 | 15.1 | 17.4 | 12.9 | 7.0 | 10.6 | 8.9 |
| **HET**  (*n=*16-17)  **vs KO**  (*n=*12-13) | <0.0001  *** | <0.0001  *** | <0.0001  *** | 0.0004  *** | 0.0002  *** | 0.0047  ** | 0.0455  * | 0.0281  * | >0.999  ns |
| delta \|dB\| | 15.8 | 16.6 | 16.7 | 12.9 | 13.6 | 10.7 | 8.2 | 8.8 | 0.9 |
| **WT**  (*n=*14-16)  **vs HET**  (*n=*16-17) | >0.999  ns | >0.999  ns | >0.999  ns | >0.999  ns | 0.6959  ns | >0.999  ns | >0.999  ns | >0.999  ns | 0.0092  ** |
| delta \|dB\| | 2.7 | 2.4 | 2.0 | 2.2 | 3.8 | 2.2 | 1.2 | 1.8 | 9.8 |

Bonferroni corrected *p* values: ****p* < 0.001; ***p* < 0.01; **p* < 0.05; ns, not significant; *n* = number of mice

**Supplementary Table 2:** Statistical comparison of click-evoked ABR wave I-IV amplitudes from GC-B WT, GC-B HET and GC-B KO mice.

| **ABR wave I amplitudes** | | | | | | | | | | | | | | | | | | | | | | | | | | | | | | | |
| --- | --- | --- | --- | --- | --- | --- | --- | --- | --- | --- | --- | --- | --- | --- | --- | --- | --- | --- | --- | --- | --- | --- | --- | --- | --- | --- | --- | --- | --- | --- | --- |
| **Stimulus level**  **(dB SL)** | **-10** | | **-5** | | | **0** | **5** | | **10** | | **15** | | **20** | | **25** | | **30** | | **35** | | **40** | | **45** | | **50** | | **55** | | **60** | **65** | **70** |
| **WT** (*n=*19-32) **vs KO** (*n=*18-31) | >0.999  ns | | >0.999  ns | | | >0.999  ns | >0.999  ns | | >0.999  ns | | 0.2098  ns | | 0.0094  ****** | | <0.0001  ******* | | <0.0001  ******* | | <0.0001  ******* | | <0.0001  ******* | | <0.0001  ******* | | <0.0001  ******* | | <0.0001  ******* | | <0.0001  ******* | <0.0001  ******* | <0.0001  ******* |
| **HET** (*n=*18-33) **vs KO** (*n=*18-31) | >0.999  ns | | >0.999  ns | | | >0.999  ns | >0.999  ns | | >0.999  ns | | 0.3469  ns | | 0.0639  ns | | 0.0011  ******* | | <0.0001  ******* | | <0.0001  ******* | | <0.0001  ******* | | <0.0001  ******* | | <0.0001  ******* | | <0.0001  ******* | | <0.0001  ******* | <0.0001  ******* | <0.0001  ******* |
| **WT** (*n=*19-32) **vs HET** (*n=*18-33) | >0.999  ns | | >0.999  ns | | | >0.999  ns | >0.999  ns | | >0.999  ns | | >0.999  ns | | >0.999  ns | | >0.999  ns | | 0.6377  ns | | 0.8076  ns | | 0.4795  ns | | 0.3358  ns | | 0.2750  ns | | 0.1224  ns | | 0.2378  ns | 0.0891  ns | 0.0132  ***** |
| **ABR wave II amplitudes** | | | | | | | | | | | | | | | | | | | | | | | | | | | | | | | |
| **WT**  (*n=*8-27) **vs KO** (*n=*12-23) | >0.999  ns | >0.999  ns | | >0.999  ns | | | >0.999  ns | | >0.999  ns | | 0.3825  ns | | 0.1153  ns | | 0.0035  ****** | | <0.0001  ******* | | <0.0001  ******* | | <0.0001  ******* | | <0.0001  ******* | | <0.0001  ******* | | <0.0001  ******* | | 0.0002  ******* | <0.0001  ******* | <0.0001  ******* |
| **HET** (*n=*15-30) **vs KO** (*n=*18-31) | >0.999  ns | >0.999  ns | | >0.999  ns | | | >0.999  ns | | >0.999  ns | | >0.999  ns | | 0.3029  ns | | 0.0032  ****** | | <0.0001  ******* | | <0.0001  ******* | | <0.0001  ******* | | <0.0001  ******* | | <0.0001  ******* | | <0.0001  ******* | | <0.0001  ******* | <0.0001  ******* | <0.0001  ******* |
| **WT**  (*n=*8-27) **vs HET** (*n=*15-30) | >0.999  ns | >0.999  ns | | >0.999  ns | | | >0.999  ns | | >0.999  ns | | >0.999  ns | | >0.999  ns | | >0.999  ns | | >0.999  ns | | >0.999  ns | | >0.999  ns | | >0.999  ns | | >0.999  ns | | 0.7584  ns | | 0.2268  ns | 0.1610  ns | 0.1449  ns |
| **ABR wave III amplitudes** | | | | | | | | | | | | | | | | | | | | | | | | | | | | | | | |
| **WT** (*n=*16-32) **vs KO** (*n=*18-31) | >0.999  ns | | >0.999  ns | | >0.999  ns | | | >0.999  ns | | >0.999  ns | | >0.999  ns | | >0.999  ns | | 0.6248  ns | | 0.1776  ns | | 0.0975  ns | | 0.0426  ***** | | 0.0026  ****** | | 0.0001  ******* | | <0.0001  ******* | <0.0001  ******* | <0.0001  ******* | <0.0001  ******* |
| **HET** (*n=*18-33) **vs KO** (*n=*18-31) | >0.999  ns | | >0.999  ns | | >0.999  ns | | | >0.999  ns | | >0.999  ns | | >0.999  ns | | >0.999  ns | | >0.999  ns | | 0.4091  ns | | 0.2441  ns | | 0.0735  ns | | 0.0070  ****** | | 0.0008  ******* | | 0.0008  ******* | 0.0012  ******* | <0.0001  ******* | <0.0001  ******* |
| **WT** (*n=*16-32) **vs HET** (*n=*18-33) | >0.999  ns | | >0.999  ns | | >0.999  ns | | | >0.999  ns | | >0.999  ns | | >0.999  ns | | >0.999  ns | | >0.999  ns | | >0.999  ns | | >0.999  ns | | >0.999  ns | | >0.999  ns | | >0.999  ns | | 0.8749  ns | 0.5705  ns | >0.999  ns | >0.999  ns |
| **Table ABR wave IV amplitudes** | | | | | | | | | | | | | | | | | | | | | | | | | | | | | | | |
| **WT** (*n=*17-32) **vs KO** (*n=*18-31) | >0.999  ns | | >0.999  ns | | >0.999  ns | | | >0.999  ns | | >0.999  ns | | 0.4212  ns | | 0.1429  ns | | 0.0639  ns | | 0.0340  ***** | | 0.0024  ****** | | 0.0007  ******* | | 0.0011  ****** | | 0.0436  ***** | | 0.1613  ns | 0.2914  ns | 0.0713  ns | 0.0288  ***** |
| **HET** (*n=*18-33) **vs KO** (*n=*18-31) | >0.999  ns | | >0.999  ns | | >0.999  ns | | | >0.999  ns | | >0.999  ns | | >0.999  ns | | 0.4123  ns | | >0.999  ns | | 0.3436  ns | | 0.6301  ns | | 0.3128  ns | | 0.1288  ns | | 0.8820  ns | | >0.999  ns | >0.999  ns | 0.4124  ns | 0.0703  ns |
| **WT** (*n=*17-32) **vs HET** (*n=*18-31) | >0.999  ns | | >0.999  ns | | >0.999  ns | | | >0.999  ns | | >0.999  ns | | >0.999  ns | | >0.999  ns | | 0.4028  ns | | 0.9769  ns | | 0.0942  ns | | 0.1058  ns | | 0.3348  ns | | 0.4405  ns | | 0.3348  ns | 0.8676  ns | >0.999  ns | >0.999  ns |

Bonferroni corrected *p* values: ****p* < 0.001; ***p* < 0.01; **p* < 0.05; ns, not significant; *n* = number of ears

**Supplementary Table 3:** Statistical comparison of click-evoked ABR wave I-IV latencies from GC-B WT, GC-B HET and GC-B KO mice.

| **ABR wave I latencies** | | | | | | | | | | | | | | | |
| --- | --- | --- | --- | --- | --- | --- | --- | --- | --- | --- | --- | --- | --- | --- | --- |
| **Stimulus level**  **(dB SL)** | **0** | **5** | **10** | **15** | **20** | **25** | **30** | **35** | **40** | **45** | **50** | **55** | **60** | **65** | **70** |
| **WT** (*n=*29-32) **vs KO** (*n=*18-31) | 0.0042  ****** | 0.0034  ****** | 0.0320  ***** | 0.0302  ***** | 0.0302  ***** | 0.0857  ns | 0.1976  ns | 0.0820  ns | 0.1976  ns | 0.0340  ***** | 0.0123  ***** | 0.0041  ****** | 0.0050  ****** | 0.0005  ******* | 0.0002  ******* |
| **HET** (*n=*31-33) **vs KO** (*n=*18-31) | 0.0386  ***** | 0.0786  ns | 0.4144  ns | 0.4144  ns | >0.999  ns | 0.7980  ns | 0.7980  ns | >0.999  ns | 0.8088  ns | 0.0860  ns | 0.2045  ns | 0.0343  ***** | 0.0373  ***** | 0.0018  ****** | 0.0050  ****** |
| **WT** (*n=*29-32) **vs HET** (*n=*31-33) | >0.999  ns | 0.8102  ns | 0.7980  ns | 0.7864  ns | 0.1852  ns | 0.7980  ns | >0.999  ns | 0.4048  ns | >0.999  ns | >0.999  ns | 0.8209  ns | >0.999  ns | >0.999  ns | >0.999  ns | 0.8335  ns |
| **ABR wave II latencies** | | | | | | | | | | | | | | | |
| **WT**  (*n=*8-27) **vs KO** (*n=*12-23) | <0.0001  ******* | 0.0001  ******* | 0.0002  ******* | 0.0028  ****** | 0.0061  ****** | 0.0075  ****** | 0.0328  ***** | 0.1133  ns | 0.0627  ns | 0.2030  ns | 0.4654  ns | 0.6530  ns | >0.999  ns | 0.5573  ns | 0.2984  ns |
| **HET** (*n=*15-30) **vs KO** (*n=*12-23) | <0.0001  ******* | 0.0202  ***** | 0.0202  ***** | 0.0715  ns | 0.2116  ns | 0.3543  ns | 0.3543  ns | 0.7925  ns | 0.8029  ns | >0.999  ns | 0.8328  ns | 0.8255  ns | >0.999  n.s | 0.3498  ns | 0.1568  ns |
| **WT**  (*n=*8-27) **vs HET** (*n=*15-30) | >0.999  ns | 0.3049  ns | 0.4690  ns | 0.7122  ns | 0.4690  ns | 0.3049  ns | 0.7573  ns | 0.7925  ns | 0.5341  ns | 0.5681  ns. | >0.999  ns | >0.999  ns | >0.999  ns | >0.999  ns | >0.999  ns |
| **ABR wave III latencies** | | | | | | | | | | | | | | | |
| **WT** (*n=*29-32) **vs KO** (*n=*18-31) | <0.0001  ******* | <0.0001  ******* | <0.0001  ******* | <0.0001  ******* | <0.0001  ******* | <0.0001  ******* | <0.0001  ******* | <0.0001  ******* | 0.0002  ******* | 0.0002  ******* | 0.0002  ******* | 0.0005  ******* | 0.0042  ****** | 0.0010  ******* | 0.0011  ****** |
| **HET** (*n=*29-33) **vs KO** (*n=*18-31) | 0.0002  ******* | <0.0001  ******* | 0.0002  ******* | 0.0002  ******* | 0.0013  ****** | 0.0013  ****** | 0.0025  ****** | 0.0025  ****** | 0.0047  ****** | 0.0008  ******* | 0.0015  ****** | 0.0031  ****** | 0.0329  ***** | 0.0168  ***** | 0.0085  ****** |
| **WT** (*n=*29-32) **vs HET** (*n=*29-33) | 0.0540  ns | 0.1713  ns | 0.4668  ns | >0.999  ns | >0.999  ns | >0.999  ns | >0.999  ns | >0.999  ns | >0.999  ns | >0.999  ns | >0.999  ns | >0.999  ns | >0.999  ns | >0.999  ns | >0.999  ns |
| **ABR wave IV latencies** | | | | | | | | | | | | | | | |
| **WT** (*n=*29-32) **vs KO** (*n=*18-31) | <0.0001  ******* | <0.0001  ******* | <0.0001  ******* | <0.0001  ******* | <0.0001  ******* | <0.0001  ******* | <0.0001  ******* | <0.0001  ******* | <0.0001  ******* | <0.0001  ******* | <0.0001  ******* | <0.0001  ******* | <0.0001  ******* | <0.0001  ******* | <0.0001  ******* |
| **HET** (*n=*28-33) **vs KO** (*n=*18-31) | <0.0001  ******* | <0.0001  ******* | <0.0001  ******* | <0.0001  ******* | 0.0001  ******* | 0.0017  ****** | 0.0010  ****** | 0.0017  ****** | 0.0017  ****** | 0.0002  ******* | <0.0001  ******* | <0.0001  ******* | 0.0020  ****** | 0.0094  ***** | 0.0127  ***** |
| **WT** (*n=*29-32) **vs HET** (*n=*28-33) | 0.7618  ns | >0.999  ns | >0.999  ns | >0.999  ns | 0.6816  ns | 0.5391  ns | 0.8492  ns | 0.5391  ns | 0.4200  ns | 0.5391  ns | 0.4200  ns | 0.5391  ns | 0.7035  ns | 0.2287  ns | 0.2360  ns |

Bonferroni corrected *p* values: ****p* < 0.001; ***p* < 0.01; **p* < 0.05; ns, not significant; *n* = number of ears

**Supplementary Table 4:** Statistical comparison of ASSR input-output functions from GC-B WT, GC-B HET and GC-B KO mice.

| **SNR** | | | | | | | | | | | | | | | |
| --- | --- | --- | --- | --- | --- | --- | --- | --- | --- | --- | --- | --- | --- | --- | --- |
| **Stimulus level**  **(dB SL)** | **-10** | **-5** | **0** | **5** | **10** | **15** | **20** | **25** | **30** | **35** | **40** | **45** | **50** | **55** | **60** |
| **WT** (*n=*8) **vs KO** (*n=*8) | >0.999  ns | 0.9130  ns | >0.999  ns | >0.999  ns | 0.0082  ****** | 0.0151  ***** | 0.2673  ns | 0.0080  ****** | 0.0768  ns | 0.0457  ***** | 0.5337  ns | 0.0626  ns | 0.2362  ns | 0.1022  ns | 0.2642  ns |
| **HET** (*n=*10) **vs KO** (*n=*8) | >0.999  ns | 0.8519  ns | >0.999  ns | 0.6881  ns | 0.1136  ns | 0.0301  ***** | 0.6406  ns | 0.0234  ***** | 0.2324  ns | >0.999  ns | 0.3176  ns | 0.1089  ns | 0.0815  ns | 0.1864  ns | 0.2140  ns |
| **WT** (*n=*8) **vs HET** (*n=*10) | >0.999  ns | >0.999  ns | >0.999  ns | >0.999  ns | 0.8224  ns | >0.999  ns | >0.999  ns | >0.999  ns | >0.999  ns | 0.2812  ns | >0.999  ns | >0.999  ns | >0.999  ns | >0.999  ns | >0.999  ns |

Bonferroni corrected *p* values: ****p* < 0.001; ***p* < 0.01; **p* < 0.05; ns, not significant; *n* = number of mice
